# Systematic in vitro evolution in *Plasmodium falciparum* reveals key determinants of drug resistance

**DOI:** 10.1126/science.adk9893

**Published:** 2024-11-29

**Authors:** Madeline R. Luth, Karla P. Godinez-Macias, Daisy Chen, John Okombo, Vandana Thathy, Xiu Cheng, Sindhu Daggupati, Heledd Davies, Satish K. Dhingra, Jan M. Economy, Rebecca C. S. Edgar, Maria G. Gomez-Lorenzo, Eva S. Istvan, Juan Carlos Jado, Gregory M. LaMonte, Bruno Melillo, Sachel Mok, Sunil K. Narwal, Tolla Ndiaye, Sabine Ottilie, Sara Palomo Diaz, Heekuk Park, Stella Peña, Frances Rocamora, Tomoyo Sakata-Kato, Jennifer L. Small-Saunders, Robert L. Summers, Patrick K. Tumwebaze, Manu Vanaerschot, Guoqin Xia, Tomas Yeo, Ashley You, Francisco-Javier Gamo, Daniel E. Goldberg, Marcus C.S. Lee, Case W. McNamara, Daouda Ndiaye, Philip J. Rosenthal, Stuart L. Schreiber, Gloria Serra, Jair Lage De Siqueira-Neto, Tina S. Skinner-Adams, Anne-Catrin Uhlemann, Nobutaka Kato, Amanda K. Lukens, Dyann F. Wirth, David A. Fidock, Elizabeth A. Winzeler

**Affiliations:** 1 Department of Pediatrics, University of California San Diego; La Jolla, CA 92093, USA; 2 Department of Microbiology and Immunology, Columbia University Irving Medical Center; New York, New York 10032, USA; 3 Center for Malaria Therapeutics and Antimicrobial Resistance, Columbia University Irving Medical Center; New York, New York 10032, USA; 4 Global Health Drug Discovery Institute; Beijing, 100192, China; 5 Wellcome Sanger Institute, Wellcome Genome Campus, Hinxton, CB10 1SA, United Kingdom; 6 Biological Chemistry and Drug Discovery, Wellcome Centre for Anti-Infectives Research, University of Dundee, Dundee, DD1 5EH, United Kingdom; 7 Global Health Medicines R&D, GSK; Tres Cantos, Madrid 28760, Spain; 8 Department of Internal Medicine, Division of Infectious Diseases, Washington University School of Medicine; Saint Louis, MO 63130, USA; 9 Department of Molecular Microbiology, Washington University School of Medicine; Saint Louis, MO 63130, USA; 10 Chemical Biology and Therapeutics Science Program, Broad Institute; Cambridge, MA 02142, USA; 11 Division of Infectious Diseases, Department of Medicine, Columbia University Irving Medical Center; New York, New York 10032, USA; 12 Química Farmacéutica, Departamento de Química Orgánica, Facultad de Química, Universidad de la República; Montevideo, Montevideo CC1157, Uruguay; 13 Department of Protozoology, Nekken Institute for Tropical Medicine, Nagasaki University; Nagasaki, 852-8523, Japan; 14 Department of Immunology & Infectious Diseases, Harvard T.H. Chan School of Public Health; Boston, MA 02115, USA; 15 Infectious Disease and Microbiome Program, The Broad Institute; Cambridge, MA 02142, USA; 16 Infectious Diseases Research Collaboration, Kampala, Uganda; 17 Department of Chemistry, The Scripps Research Institute; La Jolla, CA 92037, USA; 18 Calibr, a division of The Scripps Research Institute; La Jolla, CA 92037, USA; 19 Centre International de Recherche et de Formation en Génomique Appliquée et de Surveillance Sanitaire (CIGASS), Dakar, Senegal; 20 Department of Medicine, University of California San Francisco; San Francisco, CA 94115, USA; 21 Broad Institute of Harvard and MIT; Cambridge, MA 02142, USA; 22 Skaggs School of Pharmacy and Pharmaceutical Sciences, University of California San Diego; La Jolla, CA 92037, USA; 23 Griffith Institute for Drug Discovery, Griffith University; Nathan, Queensland 4111, Australia

## Abstract

Surveillance of drug resistance and the discovery of novel targets, key objectives in the fight against malaria, rely on identifying resistance-conferring mutations in *Plasmodium* parasites. Current approaches, while successful, require laborious experimentation or large sample sizes. To elucidate shared determinants of antimalarial resistance that can empower in silico inference, we examined the genomes of 724 *Plasmodium falciparum* clones, each selected in vitro for resistance to one of 118 compounds. We identified 1,448 variants in 128 recurrently mutated genes, including drivers of antimalarial multidrug resistance. In contrast to naturally occurring variants, those selected in vitro are more likely to be missense or frameshift, involve bulky substitutions, and occur in conserved, ordered protein domains. Collectively, our dataset reveals mutation features that predict drug resistance in eukaryotic pathogens.

## One-Sentence Summary

Through the analysis of 724 genomes evolved under compound pressure, our study reveals features of drug resistance mutations in the malaria-causing parasite *Plasmodium falciparum* and forms the basis for predicting which genetic alleles are likely to confer drug resistance in malaria parasites and other pathogens.

## Main Text:

As drug resistance remains a major concern in controlling malaria, an infectious disease caused by protozoan *Plasmodium* parasites, detection of resistance is of vital importance. Because large-scale phenotyping of parasites for drug resistance is impractical and results of therapeutic efficacy studies are limited, a major goal of sequencing clinical isolates is to identify the emergence of genetic markers of drug resistance, thus providing an early warning to inform region-specific malaria treatment policies. As a result, surveillance efforts have now placed whole-genome sequences of more than 20,000 isolates of *Plasmodium falciparum*, the most common and virulent malaria parasite, into the public domain (1). A key challenge in these efforts is distinguishing functional variants, which drive the observed phenotype, from passenger mutations, i.e. those that do not confer phenotypic change. While previous studies have identified some clinical resistance markers, the identification of novel resistance alleles either in laboratory-adapted or field isolates remains inefficient.

Since the earlier use of genetic crosses to determine key genes mediating parasite resistance to pyrimethamine, chloroquine, and other antimalarials, two alternative approaches for understanding drug resistance have emerged: population-based association studies of sequenced isolates, and in vitro evolution of resistant parasites (2). Leveraging whole-genome genotyping, these approaches have enabled the identification of mutations underlying resistance to many clinical drugs and new antimalarial compounds under development. For example, genome-mapping studies first associated a >239 kilobase (kb) region surrounding the *kelch13* locus with partial resistance to artemisinins, the fast-killing components of current first-line treatments for *P. falciparum* malaria (3). Subsequent laboratory-based selection studies identified single-nucleotide variants in *kelch13* as alleles mediating resistance (4). However, both population-based association studies and in vitro resistance selection are laborious and time-consuming, limiting their utility for genomic surveillance. For example, evolving drug-resistant parasites may take several weeks or months, and the acquisition and phenotyping of the large quantity of clinical isolates needed for association studies has significant logistical challenges (1, 5). In addition, with genetic crosses (6) or genome-wide association studies (7), loci corresponding to dozens of potential phenotype-driving variants may be identified. An advantage of in vitro evolution studies is that because relatively few mutations emerge during drug selection, variants with a high probability of conferring resistance can be discovered through careful genome analysis of only a handful of independently derived drug-resistant clones. As a result, hundreds of laboratory-evolved organism genome sequences have been published. While studies of malaria parasites are among the most advanced for this type of work, the approach is also becoming widespread for other important microbes, including *Candida albicans* (8), *Mycobacterium tuberculosis* (9), and the model organism *Saccharomyces cerevisiae* (10).

To explore the potential of in silico approaches for predicting antimalarial resistance mediators by leveraging insights from in vitro evolution, we comprehensively analyzed whole-genome sequences of 724 *P. falciparum* compound-selected clones, including 210 created specifically for this work. We performed meta-analyses across compound-selected mutations to identify characteristics of functional variants associated with resistance. In addition to revealing new resistance alleles and genes, our analysis identified protein and genome features associated with drug resistance and specific protein domains that consistently yield resistance. We found that mutations in non-coding regions or the non-core genome (subtelomeric and internal regions comprised of hypervariable gene families such as *var*, *rifin*, and *stevor* (11)) seldom contribute to resistance phenotypes. Our dataset provides a starting collection for algorithms that can identify genomic changes that are likely associated with drug resistance and presents insights for distinguishing functional from nonfunctional variants in forward genetic approaches.

## Results

### Compound-selected clones contain few mutations.

We first sought to assess whether most of the nonsynonymous mutations in compound-selected clones were functional. Utilizing laboratory resources of the MalDA Consortium (5), we compiled a set of 724 whole-genome sequences from in vitro evolved *P. falciparum* strains and their isogenic parents (Data S1). Blood-stage parasites were exposed to one of 118 small-molecule growth inhibitors, ranging from tool compounds identified by phenotypic screening as reviewed in (12), licensed antimalarials, and compounds in the developmental pipeline (Data S2), for periods ranging from weeks to more than two years. These 118 compounds were collapsed into 95 groups based on shared chemical groups. Although some datasets were previously published, this work contains the meta-analysis of our entire repository of previously reported samples, samples downloaded from the NCBI Sequence Read Archive (SRA), and 210 new genomes of parasites resistant to 33 compounds (Data S1).

Clones with phenotypic data (*n* = 448) were on average 56-fold (ranging from 1.1- to 2,654-fold) more resistant to their respective compound than the matched parent (Data S1). Although sequenced in multiple locations, genome sequences were acquired using similar paired-end short-read whole-genome sequencing (WGS) methods on Illumina platforms, with an average coverage of 135×. Most clones were selected from the Dd2 (*n* = 340), Dd2-polδ (*n* = 35) or 3D7 (*n* = 328) strains, with a few derived from 7G8, NF54 or W2 (**Methods**). In some cases, multiple clones were isolated from the same selection flask and proved genetically identical; these were treated as technical replicates.

To standardize identification of mutations that evolved during selection, we aligned sequencing reads to the *P. falciparum* 3D7 reference genome. To identify high-quality variants, we applied hard filtering on single nucleotide variants (SNVs) and insertion-deletion variants (indels) according to GATK recommendations (13). Because the *P. falciparum* genome is haploid in asexual blood stages and most samples were expected to be clonal, we retained variants for which 90% or more of read calls mapped to alternate (non-reference) alleles. Variants were then assigned predicted effects using the annotation tool SnpEff (14) (Fig. S1). Because each evolved sequence came with an isogenic parent, we subtracted variants present in the parent that had not emerged over the course of compound selection. Altogether, 5,560 SNVs and indels were identified across the dataset (Fig. S1), with an average of 17 mutated genes shared within individual compound chemotype groups. Of these variants, 4,105 were in the “core” genome, which excludes genomic regions with repetitive sequences (11) (Data S3). Each clone contained, on average, 7.65 mutations relative to its parent, of which an average of 1.2 were missense mutations in core regions. Given that there are 5,247 core genes in the genome, the data show remarkable specificity and suggest that a large proportion of the identified mutations play some role in compound resistance.

### Compound-selected mutations differ from passenger mutations.

**Fig. 1.**
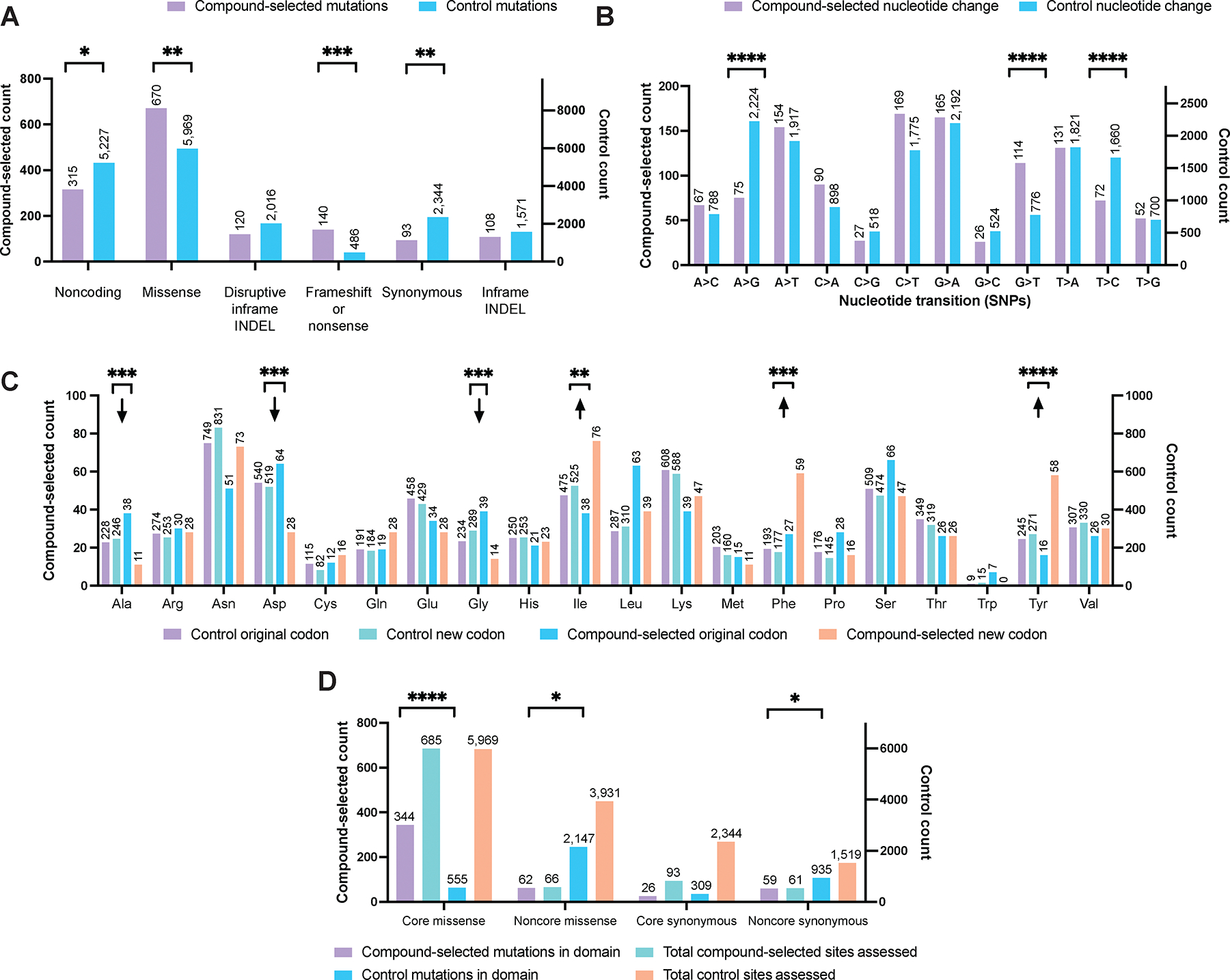
Compound-selected single nucleotide variants (SNVs) and indels have different characteristics compared to naturally occurring variants. **(A-D)** Comparison between the set of 1,448 compound-selected variants (SNVs and indels) in the core genome from this study (left axis) and a set of 17,613 control variants obtained by aligning Dd2 to the 3D7 reference genome. Axes have been normalized to display differences in proportions of different categories for compound-selected (left) and control (right) variants. **(B)** Reference base and alternate base for 1,141 unique core SNVs (coding and noncoding) compared to 15,793 control variants. **(C)** Analysis of amino acid changes for 640 core missense and 6,600 core missense control SNVs with arrows showing relative increases or decreases. **(D)** 905 evolved and 13,763 control protein coding variants were analyzed for location within a defined InterPro domain obtained from PlasmoDB v61. In all **** indicates *P* < 0.0001; *** < 0.001 and ** < 0.01 calculated using a Chi-square test.

The characteristics of our compound-selected set of SNV/indel mutations were compared to a set of control SNVs/indels that differ between two non-compound-selected clonal strains. Removing technical duplicates from the compound-selected set reduced the number to 2,628 independently derived mutations, of which 1,448 were in the core genome (Fig. S1). For the control set, we compared two laboratory strains, Dd2 and 3D7, originally derived from Southeast Asia and sub-Saharan Africa, respectively. Comparisons between Dd2 and 3D7 sequences using an identical pipeline and filters resulted in 48,364 variants, of which 38,064 were core variants. Overall, our compound-selected variants were proportionally more likely to be missense or frameshift mutations (Fig. 1A), while variants distinguishing the control clonal isolates were more likely to be synonymous. In terms of nucleotide changes, compound-selected mutations were more likely to be G to T transitions (Fig. 1B). Our data suggest that the compounds used in selections were generally not mutagenic, although there could be exceptions. The distribution of amino acid changes was also distinct from that of the control set; transitions were more likely to be from a small to a bulky amino acid such as phenylalanine or tyrosine (Fig. 1C). Finally, we examined whether core mutations were likely to be in a recognized protein domain. Around half of the core missense mutations (*n* = 685) in the compound-selected dataset were located within an InterPro protein domain. By contrast, less than 10% of the 5,969 core missense control mutations (*n* = 555) were in a recognized protein domain (Fig. 1D). These differences in distributions were significant (*P* < 0.001, hypergeometric test), highlighting that a substantial proportion of compound-selected mutations are likely to play a functional role and are unlikely to be the result of random genetic drift.

### Copy number variants frequently drive compound resistance.

**Fig. 2.**
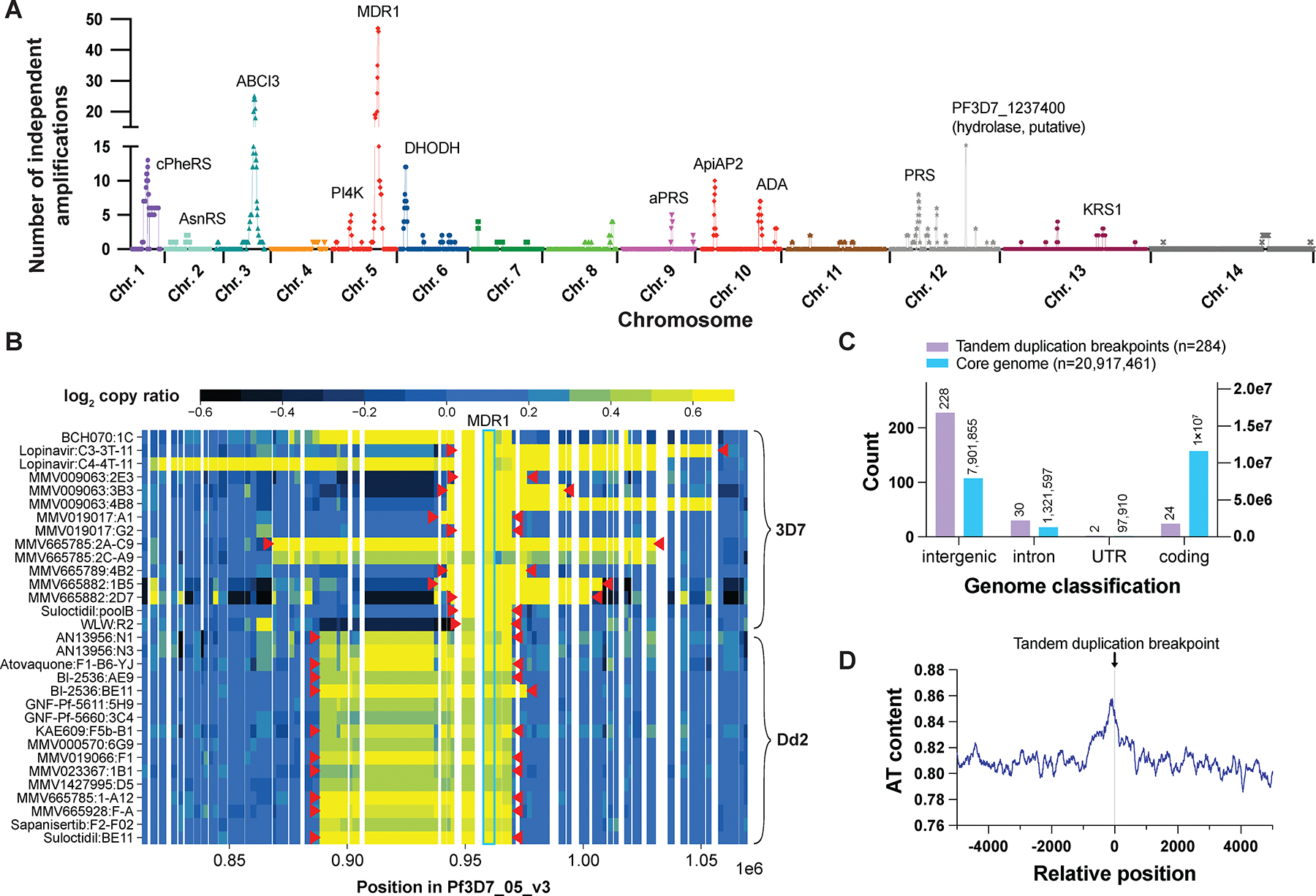
Copy number variants (CNVs) are a frequent driver of antimalarial compound resistance. **(A)** Manhattan plot showing the number of times each gene was amplified as part of an independent CNV in our dataset. Genes that are likely drivers of the selective advantage conferred by CNVs are annotated, including compound targets and multidrug resistance genes. **(B)** Heatmap visualization of amplifications containing *pfmdr1* for evolved clones (*pfmdr1* CNVs that independently involved in the same compound selection were omitted for brevity). Denoised log_2_ gene copy ratios are plotted for each clone; a contiguous segment of high log_2_ copy ratio (yellow) suggests an amplification including those genes. For CNVs identified as tandem duplications, more precise CNV boundaries are indicated by red markers. **(C)** Comparison of genomic classification distribution between 284 independent tandem duplication CNV breakpoints and all sites in the core genome. **(D)** AT content around tandem duplication breakpoints, averaged over the 284 independent tandem duplications. AT content for each relative position was computed as the proportion of bases that are A or T over all sequences beginning at that position with a sliding window of five bp. The plot is shown as a running average with a window of 100 bp.

Copy number variants (CNVs) have been shown to mediate clinically relevant drug resistance phenotypes in malaria parasites (15). To identify CNVs in our WGS dataset, we calculated differences in read coverage for core genes and compared coverage to that of a panel of untreated controls assembled from parent clones, depending on whether reads were derived from the 3D7 or Dd2 strain background (Data S4, Fig. S2). To identify the amplified or deleted sequence region comprising the CNV, we identified groups of four or more genes with log_2_ > 0.4-fold change in coverage relative to their parent strain. This resulted in 1,168 potential amplification events. Using a Kruskal-Wallis test comparing copy ratios of genes within the amplification boundaries to those averaged across the corresponding panel of controls, we found that 271 of the 724 clones harbored at least one of 420 CNVs with *P* < 0.0001. These CNVs included amplifications of known targets, such as *pfpi4k*, *pfproRS*, and the acetyl-CoA transporter, as well as multidrug resistance genes (*pfabci3*, *pfmdr1*) (Fig. 2A). We also identified multiple CNVs, including one on chromosome 14, that had been noted as emerging after long term exposure to artemisinin (16). Amplifications of GTP cyclohydrolase I (*pfgch* on chromosome 12, PF3D7_1224000) were not generally included in this set, as many strains have more than one copy of *pfgch* (17).

While large CNVs were identified with this method, we noticed multiple false-positive small CNVs, especially in clones that showed high levels of gene-to-gene read coverage variation. Because removing small CNV calls might miss functionally important CNVs, we used an orthogonal, non-coverage-based CNV validation approach that took advantage of paired-end sequencing. Paired-end reads show mapping inconsistencies when located near boundaries of structural variants and can give clues about inverted or tandem duplication events (Fig. S3). Additionally, paired-end reads near recombination breakpoints may show variation in predicted inserts, with sizes similar to that of the CNV (e.g., 20 kb, instead of 1 kb). Paired-end read support as measured by a heuristic algorithm (**Methods**) or strong copy ratio support, along with confirmation by the structural variant discovery tool DELLY (18), was found for 367 of the original 1,168 amplification calls, of which 243 CNVs were independent. About half of the unsupported CNVs were in samples with the highest variability in log_2_ copy ratio across genes (the average interquartile range of log_2_ copy ratios for five samples in the MMV006901 selections was 0.41, compared to a median interquartile range of 0.18 across 3D7 samples containing at least one supported CNV). The other half tended to be in clones isolated from parents distinct from those used to denoise read counts in our CNV analysis pipeline, highlighting the need to keep a carefully matched parent clone for ratio-based methods.

To infer the gene within a CNV that confers a selective advantage, we looked for evidence of a known target or resistance mechanism within the amplified segment or for recurring CNVs and identified the gene that was amplified most frequently across the dataset in overlapping CNVs. *Pfmdr1* was amplified in 47 independent CNV events (Fig. 2A) and harbored 19 independent SNVs, making it a clear driver of resistance. For smaller CNVs, there was more ambiguity. A set of five genes on chromosome 12 was amplified 11 times, but of this set, only *pfprs* (PF3D7_1213800, proline-tRNA ligase) also contained SNVs. In several cases, there was no clear candidate resistance mediator, including a recurring CNV on chromosome 1 and one on chromosome 9. Not all CNVs are related to compound pressure. Many evolved strains contained amplification of an 86 kb region on chromosome 10 that was also found in some parent strains, including 3D7; it may contain unidentified fitness-conferring factors.

Characterization of amplification CNVs by structural variant type and breakpoint properties showed several interesting features. Paired-end read data showed that 179 of 243 independent amplifications were tandem duplications, as opposed to inverted or interchromosomal duplications. We did not find evidence that specific genomic locations are strongly preferred for amplification of driver genes, as many CNVs had different endpoints even for the same compound. For example, CNVs with varying size and breakpoint locations containing *pfmdr1* were found in resistant clones evolved from the 3D7 parent strain, which contains a single copy of *pfmdr1* (Fig. 2B). By contrast, all clones that had further amplification of *pfmdr1* compared with the Dd2 parent strain, which already has multiple copies, had similar breakpoints. Many of the amplifications were tandem duplications, consistent with the original *pfmdr1* CNV in Dd2. CNV breakpoints were determined, with nearly single base resolution, using discordant read pairs for 142 high-confidence tandem duplications. These breakpoints showed an enrichment of duplication recombination sites in intergenic regions, which typically have lower sequencing coverage (Fig. 2C). Tandem duplication recombination sites often occurred in highly AT-rich and/or repetitive regions, supporting the hypothesis that long A/T tracks present throughout the *P. falciparum* genome facilitate microhomology-mediated repair as a major mechanism of increasing copy number (19) (Fig. 2D).

### Overrepresented genes impact compound resistance and culture adaptation.

**Fig. 3.**
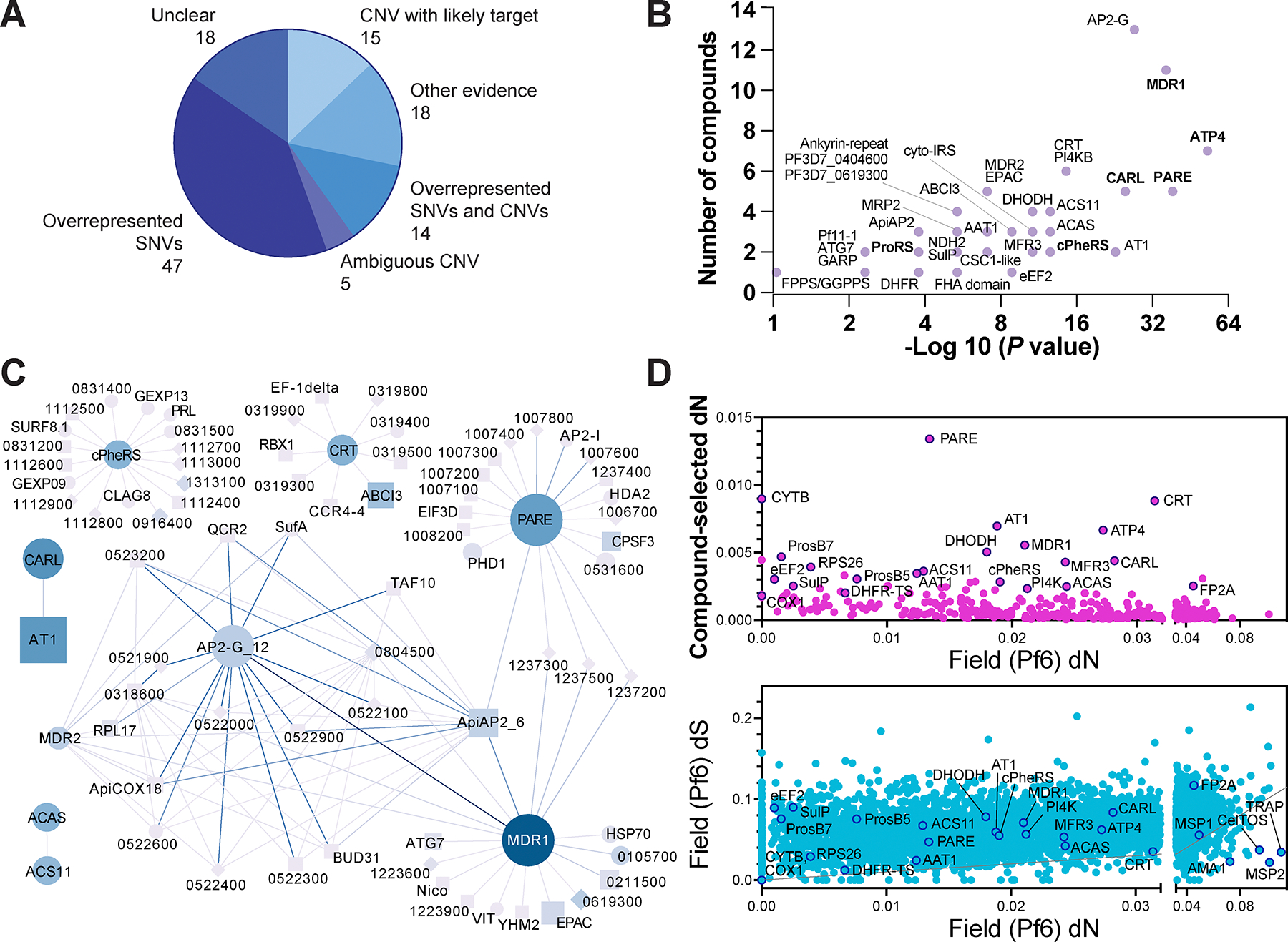
Enriched genes mutated in compound resistance selections. **(A)** Classification of evidence types supporting assignment of target or resistance mediator for 118 compounds (Data S5). “Other evidence” indicates the target/resistance gene was identified but its representation did not reach statistical significance (hypergeometric test); other experimental evidence was required for confirmation. “Ambiguous CNVs” were cases in which a CNV was found in a majority of selected clones but a clear target or resistance gene was not identified. **(B)** Enrichment P values (hypergeometric mean function) for genes that were recurrently mutated in selections for a given compound (Table S1). Structures of compounds that gave rise to SNV/indel mutations in select genes are shown in Fig. S7, including the Tanimoto chemical similarity score. Not all compounds are shown for *pfatp4* due to undisclosed structure. **(C)** Condensed version of a network linking genes for which the same pair of mutations arose in distinct compounds, filtered to show gene pairs with at least ten shared variant pair-compound pair occurrences (full network shown in Fig. S6). In cases of more than one variant pair-compound pair existing between two genes, this multiplicity was encoded as edge weight. All seven digit gene IDs have PF3D7_ removed. Circles represent known proteins; diamonds, conserved proteins; squares, putative proteins. Node size is proportional to node degree and edge color maps to occurrence of disruptive mutations from low (light blue) to high (dark blue) after adjustment for non-missense mutations. **(D)** dN for 449 genes that had more than one non-singleton SNV in the compound-selected dataset plotted against dN among Pf6 samples (top) and dS vs. dN of 4,938 core genes in the Pf6 dataset of worldwide variation (bottom).

Experimental evolution is effective at associating compounds with targets or drug resistance genes if they appear at rates higher than expected by chance. Of the 118 compounds, 47 had a target or resistance mechanism suggested by overrepresented SNVs alone, 14 by both CNVs and SNVs, and 15 by CNVs with a clear driver gene alone (Data S5). For 18 compounds, a resistance gene was determined by additional experimentation. In only 18 cases was no resistance gene identified, indicating a high success rate for experimental evolution (Fig. 3A). On a compound basis, the strongest signatures were for KAE609 (cipargamin), with 12 independent SNVs in *pfatp4* (non-SERCA-type Ca2+-transporting P-ATPase) (*P* = 9.98 × 10^−41^, Data S5), and cladosporin, showing three independent CNVs amplifying lysine-tRNA ligase (KRS1) on chromosome 13.

An advantage of this dataset is that it permits the identification of genes that appear repeatedly across disparate compounds, suggesting a multidrug resistance mechanism. Altogether, 128 genes were altered in more than one clone, 53 of which were identified three or more times. The list of statistically significant recurring genes (Table S1) contained multiple antimalarial multidrug resistance genes, such as *pfmdr1* (20) (19 independently derived SNVs/indels and 47 CNVs), chloroquine resistance transporter *pfcrt* (nine independent disruptive variants) (20), and cyclic amine resistance locus *pfcarl* (14 independent SNVs/indels and one CNV) (21). The list also contained clinically important antimalarial targets, known to acquire resistance mutations affecting inhibitor binding, such as *pfatp4* (22), *pfpi4k* (23), *pfcytb* (24), and *pfdhodh* (totaling 14 CNVs and seven SNVs) (25). Another overrepresented gene was *pfap2-g* (PF3D7_1222600), which encodes a transcription factor involved in commitment to gametocytogenesis (26), as well as the Rap guanine nucleotide exchange factor *pfepac*, both known to mutate as a result of long-term culture in the absence of drug pressure (27). Several large, conserved proteins were overrepresented, including PF3D7_0619300, PF3D7_1464500, and PF3D7_0510100, each mutated three times independently. Many of the highly overrepresented genes (7 of 14 genes mutated at least eight times independently), particularly drug targets, were also contained within CNVs. To identify genes likely to be involved in multidrug resistance, we plotted the likelihood of enrichment by chance against the number of associated compounds (Fig. 3B, Fig. S4). These data showed that genes such as *pfmdr1* and *pfap2-g* were mutated in selections with a wide variety of compounds. By contrast, targets such as *pfproRS* tended to be compound specific. This is not always the case, however, as some high-quality target genes such as *pfcytb* were identified in selections with a variety of scaffolds.

While many of these examples have been published, we also identified new gene-compound associations. For example, desoxyepothilone B is an analog of epothilone B, a naturally occurring macrolide isolated from the myxobacteria *Sorangium cellulosum*, which is known to have antitumor and antifungal activity (28). SNVs were identified in PF3D7_1008700, tubulin beta chain (Ala231Asp, Thr274Ile, *P* = 8.9 × 10^−07^). This finding supports published evidence that epothilone’s mode of action is taxol-like, inhibiting cell proliferation through microtubule stabilization (29).

GNF-Pf-5611 is an intracellular copper chelator, also known as neocuproine (30). All resistant clones selected by this compound contained at least one SNV (Lys168Ile, Ser513Leu, and Ser290*) in PF3D7_0915000, which encodes the type II NADH:ubiquinone oxidoreductase (NDH2) (Data S3, Data S5). NDH2 is one of five dehydrogenases in the *Plasmodium* mitochondrial electron transport chain that donates electrons to ubiquinone. It was considered an attractive antimalarial target until *P. falciparum* asexual blood stage (ABS) non-essentiality was shown (31). Based on its proposed mechanism, NDH2 is unlikely to be a direct target of GNF-Pf-5611. Rather, NDH2 mutations may alter interactions with the cofactor flavin adenine dinucleotide (FAD), as they either truncate NDH2 (Ser290*) or fall near its FAD-binding domains (Lys158Ile, Ser513Leu) (32) (Fig. S5). These mutations could help parasites mitigate the effects of oxidation induced by copper chelation.

Selections with MMV008434 yielded six parasite clones with low-grade resistance phenotypes (1.2- to 2.3-fold relative to the parent) (Data S1). Of these, three missense SNVs and two disruptive indels were identified in PF3D7_0609100, putatively annotated as zinc transporter *pfzip1* (Data S3, Data S5, *P* = 4.05 × 10^−19^). A PfZIP1 conditional knockout was generated (**Methods**) and assessed in dose-response assays against MMV008434 and its analog MMV011445. Results confirmed that PfZIP1 dysfunction contributes to resistance to both compounds (Fig. S6). MMV407834 is a compound from the Medicines for Malaria Venture Pathogen Box (33) with mid-nanomolar activity against asexual blood stage parasites (Data S2). Selections in both Dd2 and 3D7 strains yielded highly-resistant parasite clones (4 to 90-fold IC_50_ shifts) each containing one of four SNVs (Leu984Pro, Ser980Tyr, Asn36Ile, and Leu800Pro) in PF3D7_1250200, the putative calcium permeable stress-gated channel 1 (CSC1-like protein) (Data S1, Data S3, Data S5). This gene, which was non-mutable in a mutagenesis screen (34) and is expressed in ABS parasites (35), contains a Pfam domain (PF02714) inherent to a class of osmosensitive calcium-permeable cation channels that is conserved across eukaryotes (36).

### Network analysis reveals co-occurring mutations.

To reveal gene interactions that arise in compound selections, we constructed a network with an edge between two genes if the combination of a specific mutation (SNV, indel, or CNV) in one gene and a specific mutation in the other gene was found in selections with different compounds (Fig. 3C, Fig. S7) (**Methods**). This network was, in some cases, able to group genes by function. We identified well-known interactions, such as those between *pfat1* and *pfcarl*, or *pfacas* (PF3D7_0627800) and *pfacs11* (PF3D7_1238800) (37). The data also showed that three of six compounds that gave rise to *pfcrt* SNVs also selected for amplifications of *pfabci3* (Fig. 3C). There was a strong association between *pfap2-g* SNVs/indels and *pfmdr1* SNVs or CNVs. Of the 12 compounds yielding clones with *pfap2-g* mutations, seven also yielded *pfmdr1* mutations, and in five independent cases both *pfap2-g* and *pfmdr1* mutations were found in the same clone. Interestingly, 11 of 14 unique *pfap-2g* mutations were nonsense or frameshift, and only these loss-of-function *pfap2-g* mutations were found alongside *pfmdr1* amplification or missense SNVs in selections with the same compound. Furthermore, approximately 58% of genes on chromosome 12 with associations in the main network (7/12) were connected to both *pfmdr1* and *pfap2-g*. It may be that parasites cultured long term are likely to acquire loss-of-function mutations in *pfap2-g* because of pressure to eliminate genes causing gametocyte conversion in vitro, while *pfmdr1* point mutations arise infrequently. Another possibility is that *pfap2-g* regulates *pfmdr1*, and loss of *pfap2-g* function results in increased transcription of *pfmdr1*. Similarly, *pfapi-ap2* (PF3D7_0420300), a gene mutated in artemisinin selections (16), had associations with the conserved protein PF3D7_1324300. Another transcription factor, *pfapi-ap2* (PF3D7_0613800, ApiAP2_6 in Fig. 3C and Fig. S7), involved in the *Plasmodium* cell cycle and implicated in drug resistance evolution (38), had three associations with *pfpare* and ten with *pfmdr1*. This finding further supports the role of *pfapi-ap2* in contributing to multidrug resistance and culture adaptation in *Plasmodium*. *Pfapi2-i* (chromosome 10) was part of a frequent amplification event. Although there are few reports of transcription factors playing a role in antimalarial drug resistance, this is common in other species such as *S. cerevisiae* (10).

### Compound-selected mutations differ from naturally-occurring variants.

An important question in antimalarial drug development is whether natural parasite populations can readily attain resistance because of existing genetic variants and the plasticity of the *Plasmodium* genome. We compared our mutational set to nucleotide and amino acid substitution rates in the Pf6 dataset (39), which includes sequences of 7,113 *P. falciparum* isolates obtained from 28 countries (Data S6). SNVs that arose at least twice independently in our selections were rarely present in the Pf6 dataset (6 of 53). Genes with higher dN in the compound-selected set, measured as number of nonsynonymous SNVs normalized by nonsynonymous sites in the gene (**Methods**), tended to have low dN in Pf6. This suggests that in vitro selections typically introduced novel evolutionary pressures in genes that are highly conserved in the field, such as the proteasome β2 subunit (PF3D7_1328100), *pfdhfr*, and elongation factor 2 (PF3D7_1451100) (Fig. 3D). We also show that multidrug resistance genes, such as *pfmdr1* or *pfcrt*, were less conserved in field isolates than canonical enzymatic drug targets. Our data show that 4,418 nuclear genes with SNVs (excluding singletons) in the Pf6 dataset had no nonsynonymous SNVs among the 724 evolved clones (excluding singletons). Among these are genes involved in antigenic variation and immune response, such as *pfcsp* (PF3D7_0304600), *pfama1* (PF3D7_1133400), *pfmsp2* (PF3D7_0206800) and *pfceltos* (PF3D7_1216600), which have nonsynonymous variation in the field but were not mutated in our compound selections (40, 41) (Fig. 3D).

**Fig. 4.**
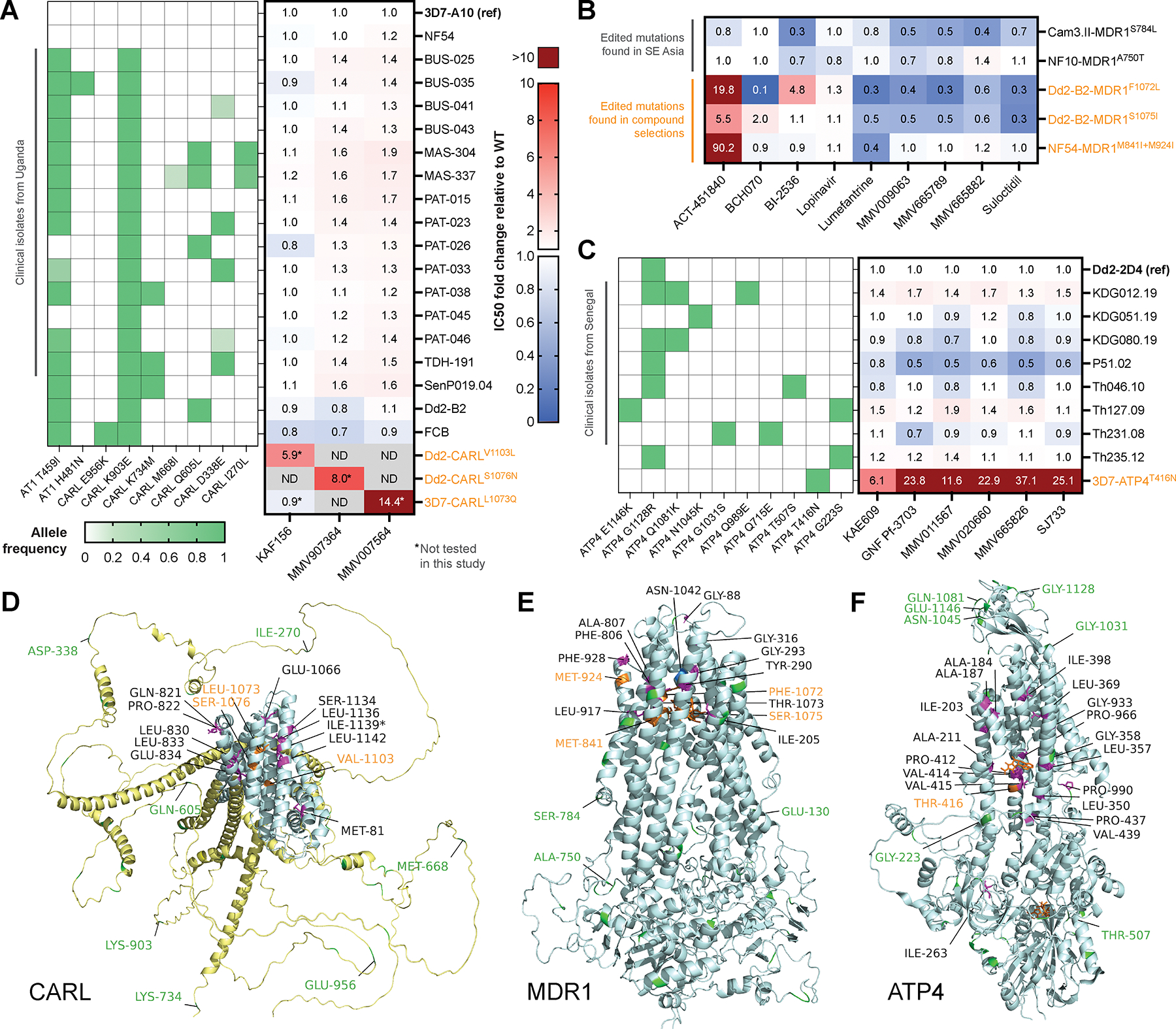
Compound susceptibility assays comparing compound-selected mutations and field variants in *pfcarl*, *pfmdr1*, and *pfatp4*. **(A)** Heatmap showing mean IC_50_ fold changes relative to 3D7-A10 from dose-response experiments for four laboratory lines and 15 clinical isolates from Uganda and Senegal with *pfcarl* variants against three compounds that select for PfCARL mutations. *Pfcarl* and *pfat1* variants were confirmed by WGS; the left heatmap shows allele frequencies for the tested lines. For reference, fold change based on previously reported IC_50_ values are shown for resistant lines with PfCARL substitutions, highlighted in orange. **(B)** Mean IC_50_ fold changes relative to isogenic parent for five edited lines with *pfmdr1* mutations. Parasite lines highlighted in orange have mutations identified from compound selections, while the rest occur in the field. **(C)** Mean IC_50_ fold changes relative to Dd2–2D4 for eight clinical isolates from Senegal and a KAE609-pressured resistant mutant, 3D7-ATP4^T416N^, highlighted in orange. *Pfatp4* allele frequencies confirmed by WGS are displayed in the left heatmap. **(D-F)** Ligand-filled models of PfCARL and PfATP4 were obtained from AlphaFill; PfMDR1 homology model was constructed with SWISS-MODEL (43) using 7a69 (see Fig. S8 for details). Mutated residues in our compound-selected dataset (with the exception of PfCARL Ile-1139, which was found in (21)) are colored magenta; mutated residues in the field with global major allele frequency (gMAF) > 0.002 were obtained from (39) and are colored green, while those found in both sets are colored blue. Labels are colored orange and green, respectively, for compound-selected vs. field mutations phenotyped in (A-C). Other compound-selected mutations are also labeled in black.

The large number of missense mutations in certain genes, such as *pfmdr1,* in both compound-selected and field samples offers the opportunity to explore whether compound-selected mutations are localized to specific protein domains. We examined the locations of compound-selected amino acid substitutions in PfCARL, PfMDR1, PfATP4, and PfCYTB (Fig. S8), all of which had ten or more independent mutations. PfCYTB and PfMDR1 mutations mediate resistance to clinical antimalarials, while mutations in PfATP4 and PfCARL mediate resistance to agents in late stages of development, namely cipargamin (KAE609) and ganaplacide (KAF156) (21, 42). We obtained protein models using either SWISS-MODEL (43) or AlphaFold (44) and mapped predicted ligands, including inhibitors, using AlphaFill. Since access to the PfMDR1 structure in Protein Data Bank (PDB) (45) was unavailable at time of the study, we used a homology model. In the PfMDR1 homology model (SMTL ID: 7a69.1) based on human ABCB1 bound to vincristine (46), all laboratory-derived mutations clustered in transmembrane domains that comprise the predicted small-molecule binding site, and virtually all mutations were located in predicted alpha helices (Fig. S8A, Fig. 4E). PfCARL is a predicted transmembrane protein and although some domains are well-conserved, thereby allowing high-confidence AlphaFold predictions (UniProt C0H483), many regions cannot be effectively folded. Notably, all 12 *pfcarl* mutations from this study are found in “ordered” domains (AlphaFold per-residue confidence score based on the lDDT structural similarity metric, pLDDT > 70), specifically predicted alpha helices (Fig. S8C, Fig. 4D), as opposed to regions predicted to be unstructured under physiological conditions (44). Likewise, in the PfATP4 model, 17 of 19 mutated residues (with the exception of Pro-990 and Ile-263) are found in alpha helices, with most located near the AlphaFill-predicted docking site of a close analog (PDB ligand ID: CZA) of cyclopiazonic acid, a potent inhibitor of the human ortholog of PfATP4, SERCA1a (Ca2+-ATPase of skeletal muscle sarcoplasmic reticulum) (47), which has 80.9% Tanimoto similarity to KAE609 (Fig. S8B, Fig. 4F). The Pro-990 mutation is observed in concert with Ile398Phe and may be compensatory. PfCYTB mutations also localized near predicted binding pockets of heme and stigmatellin, a known inhibitor of the cytochrome bc_1_ complex (48) (Fig. S8D).

We next examined the hypothesis that compound-selected missense mutations could be spatially distinguished from substitutions in *P. falciparum* field isolates. We considered SNVs with total read depth ≥ 50 and alternate allele frequency (AAF) ≥ 0.5 in at least one of the 7,113 samples in Pf6 as well as global AAF > 0.002, as calculated by summing allele depths across all Pf6 samples (Data S7). Placing the 37 highest frequency PfMDR1 missense variants in Pf6 on the homology model showed that field isolate mutations were dispersed throughout the predicted structure. Only one residue was found among both compound-selected and high-confidence field isolate mutations: Asn-1042, a variant of clinical interest (in particular, Asn1042Asp) (49) with demonstrated functional importance in modulating parasite susceptibility to 4-aminoquinoline-based drugs (50). Among less prevalent alleles in the Pf6 dataset, a small population (14 samples with total depth > 20) of field parasites from Cambodia (2011–2012) contained PfMDR1 Gly293Asp, the same residue mutated in selections with the HIV drug lopinavir (Gly293Val) and the kinase inhibitor BI-2536 (Gly293Cys) (Fig. S8A, Fig. 4E). A single Cambodian sample collected in 2008 also contained a PfMDR1 Phe806Leu, which was selected for in vitro with MMV026596. In contrast to our compound-selected mutations, which were all nonsynonymous, 28 of 65 frequently observed mutations in the Pf6 dataset encode synonymous changes. Similar results were observed for PfCARL, with all except one (Ile-1235) of the 32 high-confidence alleles from field isolates (gAAF > 0.002, at least one high-confidence sample) located in predicted disordered regions (pLDDT < 70) and none overlapping with the in vitro selected set (Fig. S8C, Fig. 4D). For PfATP4, the 40 high-confidence field mutations also appeared to be randomly distributed throughout the model (Fig. S8B, Fig. 4F). We noted a PfATP4 Gly223Ser mutation that was quite prevalent in African samples (51) and occurred at the same residue as Gly223Arg identified in selections with the spiroindolone KAE678 (22) which conferred a ~7-fold IC_50_ increase (Data S1).

### Experimental variant validation in PfMDR1, PfCARL and PfATP4.

To compare the effect of missense mutations arising from compound selections to that of naturally occurring polymorphisms, we tested the sensitivity of different PfMDR1, PfCARL and PfATP4 mutant parasites to panels of compounds. For PfCARL, 15 culture-adapted clinical isolates from Uganda and Senegal with variants relative to 3D7 and four laboratory lines (NF54, 3D7-A10, Dd2-B2 and FCB) were phenotyped against three compounds that selected SNVs in *pfcarl*: KAF156 (ganaplacide), MMV907364 and MMV007564 (Fig. 4A, Fig. S9, Table S4). Whole-genome sequencing of the 19 lines showed various missense SNVs in *pfcarl*, *pfat1*, and *pfugt*, which are additional multidrug resistance genes implicated in KAF156 and GNF179 resistance (Tables S2–3). Notably, all *pfcarl* mutations identified in the field isolates are in disordered regions of the protein’s AlphaFold structure (Fig. 4D). Mean IC_50_ fold changes compared to wildtype 3D7-A10 were small, reaching a maximum of 1.2-fold for KAF156 (for MAS-337), 1.6-fold for MMV907364 (for MAS-304, MAS-337, PAT-015 and SenP019.04), and 1.9-fold for MMV007564 (for MAS-304) (Fig. S9, Table S4; metadata shown in Table S2). In contrast, lines from resistance selections with the respective compounds with PfCARL substitutions (Val1103Leu, Ser1076Asn and Leu1073Gln) yielded substantially higher IC_50_ fold shifts of greater than five, based on previously reported IC_50_ values (52, 53) (Fig. 4A, Data S1).

For PfMDR1, we tested the sensitivities of five lines with missense mutations introduced through gene editing, and their corresponding parental lines, to eight compounds that previously yielded *pfmdr1* SNVs and/or CNVs in selections: ACT-451840, MMV665789, MMV009063, BI-2536, lopinavir, suloctidil, BCH070 and MMV665882. Lumefantrine, whose potency can be modulated by some *pfmdr1* variants, was also tested (Tables S5–7). All edited *pfmdr1* mutations were confirmed using Sanger sequencing (Fig. S10). Ala750Thr and Ser784Leu are naturally-occurring variants previously described in field isolates from Western Cambodia and the Thai-Myanmar border (54); CRISPR/Cas9 or zinc-finger nuclease (ZFN)-based editing was used to create recombinant mutants with these PfMDR1 substitutions on the NF10 and Cam3.II^C580Y^ backgrounds, respectively (**Methods**). Significant differences in mean IC_50_ and IC_90_ values across four to five biological replicates (Mann-Whitney *U* tests) between mutant lines and their isogenic parents indicate that PfMDR1 Ser784Leu increased parasite susceptibility to five of the eight tested compounds, while Ala750Thr did not influence the activity of any (Fig. 4B, Fig. S11). PfMDR1 Met841Ile+Met924Ile, identified in ACT-451840 selections and edited into NF54 parasites (55), only altered susceptibility to ACT-451840. Phe1072Leu and Ser1075Ile were identified after selection with GNF-Pf-5660 and GNF-Pf-5668, respectively, and each mutation was edited into Dd2-B2 parasites using CRISPR/Cas9 (56). Phe1072Leu conferred resistance to BI-2536 and sensitization against BCH070; by contrast, Ser1075Ile did not. Both mutations conferred cross-resistance to ACT-451840 and are positioned next to vincristine in the PfMDR1 homology model (Fig. 4E). Furthermore, BCH070 selections yielded a substitution, Thr1073Ile, near the substrate-binding domain inside the transport channel of PfMDR1 in its inward-facing conformation (45). Consistent with the hypothesis that PfMDR1 binding-domain mutations modulate substrate recognition, both Phe1072Leu and Ser1075Ile increased susceptibility to compounds that select for PfMDR1 amplifications.

Ten parasite lines, including Dd2–2D4 (a clone of Dd2), 3D7-ATP4^T416N^ (a KAE609-pressured resistant mutant (57)), and eight clinical isolates from Senegal (58) with distinct *pfatp4* genotypes, were phenotyped against 12 compounds, of which six have selected for resistant alleles in *pfatp4* (KAE609, SJ733, MMV665826, MMV020660, MMV011567, GNF-Pf-3703) while the rest are commonly used antimalarial drugs (artemether, piperaquine, amodiaquine, mefloquine, atovaquone, chloroquine) (Fig. 4C, Fig. S12, Table S10). Whole-genome sequencing was performed on all lines and clinical isolates to profile missense mutations in *pfatp4* (Tables S8–9). Only 3D7-ATP4^T416N^ showed a substantial increase in IC_50_ relative to Dd2–2D4 for the six *pfatp4*-associated compounds. While some of the field mutations are in ordered regions of the PfATP4 AlphaFold structure, only Thr416Asn is located near the AlphaFill-predicted binding site of the cyclopiazonic acid analog (PDB ligand ID: CZA), a known inhibitor of the human ortholog of PfATP4 (47) (Fig. 4F).

## Discussion

Here, we present a comprehensive dataset of compound-selected resistance alleles for the *P. falciparum* malaria parasite. Similar to MalariaGEN’s latest Pf7 dataset of field isolate sequences, we anticipate this resource and insights into shared characteristics of resistance-conferring alleles will be useful for several applications in the discovery and deployment of antimalarial drugs.

The striking breadth of resistance mechanisms across our dataset indicates that *P. falciparum* evolves resistance with relative ease. Indeed, half the compounds in our set, which encompasses much of the chemical space in the current portfolio of next-generation antimalarials, appear vulnerable to resistance acquisition. Drug development strategies should therefore minimize this liability by prioritizing resistance-refractory compounds as well as identifying collateral sensitivity pathways, in which resistance to one antimalarial increases sensitivity to another, to inform combination therapies. Analysis of recent African *P. falciparum* isolates presumed to be multidrug-resistant showed that a region on chromosome 12 containing *pfap2-g* (PF3D7_1222600) and *ap2–12* (PF3D7_1222400) (59) is under strong selection. In vitro evolved artemisinin-resistant parasites in our dataset had mutations in an AP2 transcription factor on chromosome 4 (PF3D7_0420300), as well as a chromosome 14 amplification containing an AP2 transcription factor (PF3D7_1456000) (16). PF3D7_0420300 was also mutated in strains pressured with GNF179, closely related to ganaplacide, a compound in advanced stages of development that is likely to be licensed to treat malaria, while mutations in its 5’ untranslated region were found in parasites pressured with atovaquone or the experimental compounds AN13762 and MMV024038. These data show the high level of interconnectivity among resistance mechanisms for both existing drugs and new compounds under development.

As we show, in vitro evolution of compound resistance typically gives rise to few mutations over the course of compound selection, compared to the thousands of genetic variants which distinguish even slightly diverged isolates from the field. However, it remains the case that most SNV/indel mutations in our dataset likely do not drive compound resistance and instead are neutral mutations or improve fitness in in vitro culture. Moreover, not all genes enriched for in vitro evolved mutations are drivers of compound resistance; some may play roles in culture adaptation, while multigene families in non-core hypervariable regions were also frequently mutated. Further experimental work is needed to validate the roles of these overrepresented genes.

Although transcription factor mutations appear to play a larger role in drug resistance in other microbial species, such as *S. cerevisiae* (10), the frequent appearance of *P. falciparum* mutations in ApiAP2 transcription factors indicates that transcriptional regulation may play an important role in stress adaptation in malaria parasites. We found mutations in AP2 transcription factors at higher rates than expected, especially when comparing sequences of laboratory-selected parasites to those of parasite field isolates. AP2 transcription factors were first identified in *Plasmodium* based on their similarity to the AP2 transcription factor family in plants; these proteins are now known to be key regulators of various stress responses (38, 60). Mutations in ApiAP2 genes were frequently selected in this study. One example is PF3D7_0613800, which had mutations after selections with nine different compounds but did not bear more than one independent mutation for any compound. While three of the selected PF3D7_0613800 mutations are missense, there are also inframe deletions, all in structurally disordered domains that are less likely to play a functional role. In *S. cerevisiae*, gain of function mutations in Zn(2)-C6 transcription factors *yrr1* and *yrm1* are also primarily found in distinct, structurally disordered but spatially conserved regions (10).

Importantly, our dataset can also serve to inform considerations of non-malarial resistance mechanisms. For example, the dataset can be a resource for identifying potential drug resistance alleles in microbial pathogens or cancer cells collected from patients treated with dihydroorotate dehydrogenase inhibitors, which frequently target the same ubiquinone-binding pocket in a range of organisms (61). Our work suggests that drug resistance mechanisms are often conserved across species. As an example, cladosporin resistance has been associated with lysyl-tRNA synthetase mutations in yeast, and in *Plasmodium* cladosporin selected for amplifications of lysyl-tRNA synthetase (62).

Consistent identification of specific mutations in independently evolved resistant parasites was usually sufficient to identify driver mutations, many of which were subsequently validated through genetic editing. However, in several cases the variant(s) underlying resistance remained unclear. This challenge is pronounced for selections in which many nonsynonymous mutations arose after long selection times or were identified in the hypermutable *P. falciparum* Dd2-polδ strain. Importantly, secondary contributors to antimalarial resistance, variants sequenced with low allele frequency due to multiple gene copies or non-clonality, or variants with less obvious effects such as noncoding DNA mutations or balanced structural variants could be overlooked. Improved mutation calling methods could potentially uncover additional contributors to antimalarial resistance in this dataset, and an in silico approach to variant prioritization may help elucidate resistance-conferring alleles in unresolved selections.

While an advantage of our dataset is the diversity of compounds and resistance mechanisms included, certain compounds and drug targets are overrepresented. Thus, caution must be taken to ensure that models trained on this dataset do not learn compound-specific biases. The mutations we identified also should not be interpreted as capturing all possible resistance drivers for any given parasite strain and compound, as most selections yielded only a few independent resistant clones. Further experimental work, such as continuous directed evolution (63) and minimum inoculum of resistance (MIR) studies (64), will be required to fully characterize how resistance arises against specific compounds.

There are limitations to using in vitro-selected mutations to inform drug development decisions. For instance, in vitro models of *Plasmodium* infection cannot recapitulate the selective pressures applied by the host immune system and other host-pathogen, host-vector, or host-xenobiotic interactions. Additionally, most laboratory strains of *P. falciparum* are not derived from recent parasite isolates. Rather, these parasites likely contain multiple adaptations to long-term culture, and their genetic backgrounds may not be fully representative of current natural parasite populations. Despite differences in selective pressure in the laboratory versus the field, insights from experimentally controlled compound selections are valuable.

Finally, our work reveals how to distinguish phenotype-driving variants from passengers, a key challenge of forward genetics approaches. This dataset and others could empower future machine learning-based approaches to estimate variant functionality in silico.

## Materials and Methods:

### Data acquisition

Sequencing information for parasite samples with evolved resistance to 25 antimalarial compounds were downloaded from the NCBI Sequence Read Archive (SRA) (https://www.ncbi.nlm.nih.gov/sra) or acquired through direct correspondence with the senior author(s) of published work. These compounds include the gold-standard antimalarial drug artemisinin (16); the benzoxaboroles AN13956, AN13762, AN10248 (65); the triazolopyrimidine analog series including DSM1 (25), DSM265, and DSM267 (66); the imidazolopiperazine GNF179 (52); halofuginone, a synthetic derivative of the natural quinazolinone alkaloid febrifugine (67); the boronate human proteasomal inhibitor bortezomib (68); the pantothenamide CXP18.6–052 (69); the dihydroisoquinolones SJ733, SJ247, SJ311 and SJ279 (70); the 2,6-disubstituted quinoline-4-carboxamide DDD107498 (71); the antitubercular clinical candidate SQ109 (72); the peptide vinyl sulfones WLL-vs and WLW-vs (73); the primary sulfonamide glycoside PS-3 (74); the bis-1,2,4-triazine compound MIPS-0004373 (75); MMV019313 (76); the trisubstituted imidazole MMV030084 (77); the 3-hydroxypropanamidine compound 22 (78); and the pyrazolopyrimidine sulfamate ML901 (79).

### Parasite strain information

*P. falciparum* 3D7 is a mostly drug-sensitive line, although it is partially resistant to sulfadoxine and contains an amplification of GTP cyclohydrolase (17). The 3D7-A10 clone has been used by MalDA in previous compound selection and sequencing efforts (15). Dd2 is a multi-drug resistant line originating from an Indochina III/CDC isolate that contains a mutant *pfcrt* sequence and amplifications in *pfmdr1* and GTP cyclohydrolase that confer reduced susceptibility to chloroquine, mefloquine, lumefantrine, and pyrimethamine (80). Dd2-B2 and Dd2-B4 are genetically homogenous, independently isolated lines that were cloned from Dd2 by limiting dilution (56, 81). The Dd2-polδ line is a Dd2 strain that was genetically modified at the DNA polymerase δ subunit (Asp308Ala and Glu310Ala) to disrupt proofreading function and increase accumulation of mutations over successive replication events (82). W2 is an Indochinese chloroquine-resistant line and 7G8 is a Brazilian line resistant to chloroquine, amodiaquine (partially), and pyrimethamine (83, 84). V1/S is a chloroquine-, pyrimethamine-, and cycloguanil-resistant strain from Vietnam (85). Isolates from Uganda were collected and cultured as previously described (86) and studied after culture adaptation and shipping to the USA. Isolates from Senegal were collected and culture-adapted as previously described (58).

### In vitro evolution of compound-resistant parasites

*P. falciparum* parasites were cultured in RPMI1640 media supplemented with 0.5% AlbuMAX II, 4.3% human serum, 25 mM Hepes, 25 mM NaHCO_3_, 0.36 mM hypoxanthine, and 100 ug/mL gentamicin. Cultures were maintained in leukocyte-depleted red blood cells (RBCs) at 2.5% hematocrit (HCT) and incubated at 37°C in an atmosphere of 3% O_2_, 4% CO_2_, and 93% N_2_. In some cases only AlbuMAX II supplemented RPMI media without gentamicin and with 0.15 mM hypoxanthine, or only human serum supplemented RPMI media with gentamicin and with 0.15 mM hypoxanthine, was used in standard culture conditions. The human biological samples were sourced ethically and their research use was in accord with the terms of the informed consents. Resistant parasites were generated using either a high-pressure pulse, ramp-up, stepwise, or constant method of compound exposure as previously described (15). Cultures were maintained under selection conditions until they demonstrated a reproducible IC_50_ fold-shift of at least 3×, at which point parasites were cloned in 96-well plates by limiting dilution (87).

Compound IC_50_ was assessed in dose-response format including using a SYBR Green-I based cell-proliferation assay as previously described (88). Parasites were incubated for 72 h in 96-well or 384-well plates with exposure to the compound of interest in a 12-point dilution series. An artemisinin dilution series was conducted in parallel as a positive control. Following the incubation, parasites were lysed, and DNA was stained using SYBR Green fluorescence and was measured at 535 nm on an Envision plate reader (Perkin Elmer, Waltham, MA) or SpectraMax iD5 plate reader (Molecular Devices, San Jose, CA) after excitation at 485 nm. For some lines, *P. falciparum* growth inhibition was determined using a modified in vitro [^3^H]-hypoxanthine incorporation method as previously described (24) or flow cytometry on an iQue Screener PLUS (Sartorius) with parasites stained with 1× SYBR Green and 200 nM MitoTracker Deep Red (56, 77). Dose-response curves were fitted and log(IC_50_) values calculated using Prism (GraphPad Prism, La Jolla, CA), or Excel and Grafit 5 software. IC_50_ fold-shift changes were calculated by comparing IC_50_ of the resistant clones to that of the corresponding compound-sensitive parent line.

### Whole-genome sequencing analysis and annotation of variants

Raw sequencing reads were aligned to the *P. falciparum* 3D7 reference genome (PlasmoDB v13.0) and pre-processed following standard GATK version 3.5 protocols (13). SNVs and indels were called with GATK HaplotypeCaller and filtered to retain high-quality variants. SNV calls were retained if they did not meet the following exclusion criteria: ReadPosRankSum > 8.0 or < −8.0, QUAL < 500, Quality by Depth (QD) < 2.0, Mapping Quality Rank Sum < −12.5, and filtered depth (DP) < 7. Indels were retained if they did not meet the following exclusion criteria: ReadPosRankSum < −20, QUAL < 500, QD < 2, and DP < 7. Allele fraction cutoffs for alternate alleles were ≥ 0.35 for bulk samples and ≥ 0.90 for clonal samples. Functional annotation of variants was carried out using SnpEff (14) with a custom database built from the 3D7 GFF from PlasmoDB (https://plasmodb.org/plasmo/app). Mutations that were considered background (native to the compound-sensitive parent) were removed, leaving a list of only high-quality mutations that had evolved over the course of the compound selection process.

Mutations were assessed for whether they were positioned within annotated InterPro domains, low-complexity regions as defined by the PlasmoDB Genome Browser, or 3D7 5’/3’ untranslated regions (UTRs) as defined by Chappell *et al.* 2020 (89).

CNVs were detected by calculating denoised log_2_ copy ratios across gene intervals through the GATK 4.0 CNV pipeline. We constructed two separate panels of normals for Dd2 and 3D7, using 30 independently sequenced parent clones of each genetic background. Read counts for each sample were calculated across a predefined gene interval list where intergenic regions and the highly variable *pfvar, pfrifin, pfstevor,* and *pfsurfin* genes (90) were removed. Following denoising against the strain-matched panel of normals, log_2_ copy ratios were calculated for each gene interval. CNVs were retained if at least 4 sequential genes showed a denoised log_2_ copy ratio of at least 0.4, indicating potential gene duplication.

To account for a subset of samples yielding false positives due to noisy or altered coverage profiles, we further filtered the candidate CNV list obtained from segmentation of genic copy ratios based on three additional criteria: statistical significance of difference in copy ratio vectors, discordant read-pair support for tandem duplications, and overlap with an independent CNV calling method, DELLY (18). DELLY is an integrated structural variant discovery method that utilizes both paired-end and split-read analysis to call CNVs. To assign confidence to CNVs based on the copy ratio data, Kruskal-Wallis test was performed on the copy ratio vectors of each candidate CNV and averaged copy ratio across all parent samples in the corresponding panel of normals. The resulting *P* values were used to quantify how strongly a candidate CNV’s copy ratio vector deviates from parents. As orthogonal evidence for the presence of duplications, we wrote an algorithm that searches for discordant paired end reads within a 10 kb window of candidate CNV boundaries, excluding noncore regions. This approach was effective for validating tandem duplications that produce a discordant read-pair signal at both boundaries, which is unlikely to occur multiple times by chance. While DELLY alone yielded a large number of false positives as well, sufficiently high quality DELLY calls were useful for validating candidate CNVs with strong discordant read-pair and split-read support that were not necessarily tandem duplications.

Based on manual inspection of select CNVs, we decided on a set of heuristics for filtering candidate CNVs to produce the final list. Samples were classified based on interquartile range (IQR) of core genome-wide gene copy ratios; stricter support thresholds were applied to those with high (> 0.3) or intermediate (0.12–0.3) IQR. Adjacent candidate CNVs were also combined. To optimize sensitivity and specificity on a manually validated test set, candidate CNVs were retained if they either had both low *P* value and sufficient overlap with DELLY calls, or both tandem duplication support (not near noncore regions) and some overlap with DELLY calls. The specific thresholds used in the filtering step are described in Data S4.

### Gene network construction

A subset of the variants was generated using SNV/indel (Data S3) and CNV (Data S4) data filtered for core genes and mutation types, i.e. missense, disruptive inframe insertion or deletion (indel), frameshift, start loss, stop gained or lost, splice region variant and combinations of these. The list was processed to create pairs of compound-gene(s) and compound-gene-variant type. The network was generated using these pairs, having genes as nodes, and edges where two genes have at least once instance of a pair of compounds yielding resistant samples with the same SNV/indel (gene 1)–SNV/indel (gene 2) or SNV/indel (gene 1)–CNV (gene 2) variant pair. The network was visualized using Cytoscape v.3.9.1 organic layout (91). The node color represents the score calculated based on the variant type adjusting for nonsense (light blue, lower score) to missense (dark blue, higher score); the total of variants is shown by the node’s size adjusting for CNV (smaller) vs SNV/indel (bigger). Edge intensities show the total number of mutations combined by the compound pair from light grey (less mutated) to blue (highly mutated). Highly variable multigene families (*pfvar, pfrifin, pfstevor,* and *pfsurfin)* were removed from the final network.

### Parasite culture

For the experiments testing for susceptibility to compounds (KAF156, MMV907364, and MMV007564) that select for PfCARL mutations (Figs. 4A and S6, Tables S2–4), asexual blood stage (ABS) parasites, including laboratory lines and culture-adapted field isolates, were cultured at 2% HCT in human O+ or A+ RBCs in RPMI-1640 media, supplemented with 25 mM HEPES (Fisher), 50 mg/L hypoxanthine (Sigma Aldrich), 2 mM L-glutamine (Cambridge Isotope Laboratories, Inc.), 0.21% sodium bicarbonate (Sigma Aldrich), 0.5% (wt/vol) AlbuMAX II (Invitrogen), 8% filtered, heat-inactivated, pooled off-the-clot AB+ human serum (Innovative Research, Inc.), and 10 μg/mL gentamicin (Fisher). Cultures were propagated in tissue culture flasks gassed with a mixture of 5% O_2_, 5% CO_2_ and 90% N_2_ and maintained at 37°C.

For the experiments phenotyping PfMDR1 mutant lines (Tables S5–7), clonal parasite lines were thawed from frozen stocks and put into culture where they were maintained at 3% HCT in human O+ RBCs in RPMI-1640 media as listed above. Once parasites attained 3% parasitemia, infected blood pellet was collected, and DNA was extracted for PCR and sequencing of the *pfmdr1* loci of interest to ascertain presence of the mutations at the correct codon position.

For the experiments testing for susceptibility to compounds (KAE609, SJ733, MMV665826, MMV020660, MMV011567, GNF Pf-3703) that select for PfATP4 mutations (Figs. 4C and S9, Tables S8–10), ABS parasites, including laboratory lines and culture-adapted field isolates, were cultured at 5% HCT in RPMI-1640 media as listed above, with a lower O_2_ gas mixture (1% O_2_, 4.1% CO_2_ and 94.9% N_2_).

### Targeted *pfmdr1* sequencing and analysis of mutations

The *pfmdr1* gene was PCR-amplified from parasite genomic DNA using primers flanking the edited locus in PF3D7_0523000. Genomic DNAs of parental strains were used as a control. The PCR conditions to amplify a 812 bp fragment using primer pair p9160+p9161 encompassing the Ala750Thr SNV in NF10, Ser784Leu SNV in Cam3.II, *or* Met841Ile+Met924Ile SNVs in NF54 were: 95°C for 3 minutes, 35 rounds of 95°C for 30 seconds, 58°C for 50 seconds, and 62°C for 1 minute, with a final extension of 3 minutes at 68°C. PCR conditions to amplify a 1130-base pair (bp) fragment encompassing the Phe1072Leu and Ser1075Ile SNVs in Dd2-B2 using primer pair p7823+p7923 and KAPA HiFi HotStart ReadyMix (2X) (Roche) were: 95°C for 3 minutes, 35 rounds of 95°C for 30 seconds, 52°C for 1 minute, and 62°C for 1 minute, with a final extension of 3 minutes at 68°C. Sequences of the primers are given in Table S6.

### Compound susceptibility assays for lines with *pfcarl* or *pfmdr1* mutations

To define the 50% (IC_50_) and 90% (IC_90_) growth-inhibitory KAF156, MMV907364, and MMV007564 concentration values for parasites, asynchronous ABS cultures at 0.2% to 0.8% parasitemia and 1% HCT in 0.5% (wt/vol) AlbuMAX II/8% serum-containing media were exposed for 72 h to a range of ten compound concentrations that were 2-fold (MMV007564) or 3-fold (KAF156 and MMV907364) serially diluted in duplicates along with compound-free controls. For the lines with edited *pfmdr1* mutations, asynchronous parasites at 0.2% parasitemia and 1% HCT in 0.5% (wt/vol) AlbuMAX II-containing media were exposed for 72 h to a range of ten compound concentrations that were 2-fold serially diluted in duplicates along with compound-free controls. Parasite survival was assessed by flow cytometry on an Intellicyt iQue Screener PLUS (Sartorius) or a BD FACSCelesta (BD Biosciences) using 1× SYBR Green (Invitrogen) and 200 nM MitoTracker Deep Red FM (Life Technologies) as nuclear stain and vital dyes, respectively. IC_50_ and IC_90_ values were derived from growth inhibition data using nonlinear regression (Prism 10, GraphPad) or linear interpolation (assays for lines with *pfcarl* mutations) as means ± SEM from 4 to 5 independent biological repeats with two technical replicates. Statistical significance of IC_50_ and IC_90_ shifts compared to wildtype reference line (3D7-A10 for *pfcarl*) or isogenic parents (*pfmdr1* edited lines) was determined using two-tailed Mann-Whitney *U* tests (Prism 10, GraphPad).

### Compound susceptibility assays for lines with *pfatp4* mutations

Compound IC_50_ was assessed in dose-response format including using a SYBR Green-I based cell-proliferation assay as previously described (88). In brief, cultures synchronized by sorbitol at 1% ring-stage parasitemia and 1% HCT in 0.5% (wt/vol) AlbuMAX II-supplemented media (described above) were incubated for 72 h in 384-well plates with exposure to the compound of interest in a 12-point dilution series with technical triplicates. A fixed concentration of dihydroartemisinin was used as a positive control. Compounds were dispensed with an HP D300 Digital Dispenser (Hewlett Packard, Palo Alto, CA, USA). Following the incubation, parasites were lysed, DNA was stained using SYBR Green fluorescence and was measured at 535 nm on a SpectraMax iD5 plate reader (Molecular Devices, San Jose, CA) after excitation at 485 nm. Data were archived and analyzed using the CDD Vault from Collaborative Drug Discovery (Burlingame, CA. www.collaborativedrug.com). IC_50_ fold changes were calculated by comparing IC_50_ of the *pfatp4* mutant isolates to that of the compound-sensitive Dd2–2D4 parasite line.

### Whole-genome sequencing of lines phenotyped against *pfcarl* or *pfatp4* mutation-selecting compounds and variant analysis

Parasite genomic DNA was extracted upon completion of the dose-response assays. Cultures at 5–10% parasitemia were initially lysed with 0.15% saponin and washed twice with 1× PBS. Genomic DNA was extracted using the QIAamp DNA Blood Mini Kit (Qiagen). Whole-genome sequencing (WGS) was performed using a Nextera Flex DNA library kit and multiplexed on a MiSeq flow cell to generate 300 bp paired-end reads. Sequences were aligned to the Pf3D7 reference genome (PlasmoDB, version 48) using the Burrows-Wheeler Aligner (BWA). PCR duplicates and unmapped reads were filtered out using Samtools and Picard. Base quality scores were recalibrated using GATK BaseRecalibrator. GATK Haplotype Caller (version 4.1.8) was used to identify all possible single nucleotide variants (SNVs) in test parasite lines filtered based on quality scores (variant quality as function of depth QD > 1.5, mapping quality > 40, min base quality score > 18, read depth > 5) to obtain high quality SNVs that were annotated using SnpEff. Comparative SNV analyses between the laboratory or field isolate genomes and the reference Pf3D7 genome were performed to generate the final list of SNVs. BIC-Seq was used to discover copy number variations (CNVs) against the reference strain using the Bayesian statistical model. SNVs and CNVs were visually inspected and confirmed using Integrative Genome Viewer (IGV). Low frequency SNVs (< 70% alternate allele frequency) were quantified by visual inspection of the reads covering individual loci using IGV. Alternate allele frequencies were derived from observed alternate reads / sum of alternate and reference reads.

### PfZIP1 conditional knockout

For the pZIP1-cKO repair plasmid, a recodonised sequence encompassing amino acids 242–358 (C-term) of PfZIP1 and a barcode cassette (100 bp) were ordered as gBlocks (IDT). The 5’ homology region (540 bp) and 3’ homology region (496 bp) were PCR-amplified from parasite genomic DNA. The first loxP intron and a sequence containing a 3xV5 tag, 2A skip peptide, Neomycin resistance cassette, and second loxP intron, were PCR-amplified from a previously used plasmid (92). Fragments were inserted by Gibson assembly into a pUC19 vector with an ampicillin resistance cassette. The guide RNA sequence (TGTCCTAACACTATACCCAG) was inserted into a double BbsI site within the pDC2-coCas9-gRNA plasmid (93). The repair plasmid (60 μg), the guide RNA plasmid (30 μg) and 100 μL packed NF54 DiCre (94) parasites were suspended in cytomix (120 mM KCl, 0.15 mM CaCl_2_, 2 mM EGTA, 5 mM MgCl_2_, 10 mM K_2_HPO_4_/KH2PO_4_, 25 mM HEPES, pH 7.6) and transfected using a Biorad GenePulser II electroporator (310 V, 950 μF, 200 Ω) in 2 mm electroporation cuvettes (Biorad). After 24 h the transfected parasite lines were treated with 2.5 nM WR99210 for 5 days to select for the Cas9 plasmid. Once parasites recovered these were treated for 2 weeks with 225 μg/ml G418 to select for correct integration into the PfZIP1 locus. Parasites were cloned by limiting dilution, and correct integration and rapamycin-induced excision verified by PCR.

### *Pfmdr1* gene editing

The *pfmdr1* Ala750Thr and Ser784Leu mutations were introduced into NF10 and Cam3.II^C580Y^ parasites, respectively, using a T7-based CRISPR/Cas9 two-plasmid system described previously (55). Site-directed mutagenesis using primer pairs p6985+p6986 and p6987+p6988 was used to reverse Met841Ile+Met924Ile mutations to the wild-type residues in the pT7pol-donor-bsd plasmid harboring the Met841Ile+Met924Ile *pfmdr1* donor sequence, respectively. A second round of site-directed mutagenesis (SDM) on this donor plasmid using primer pairs p6989+6990 or p6916+p6917 resulted in two donor plasmids harboring mutations Ser784Leu or Ala750Thr, respectively. Donor plasmids were co-electroporated with the pCas9-gRNA2-h*dhfr* plasmid into parasites that were cultured in the presence of 2.5 nM WR99210 and 2.0 mg/ml blasticidin. *pfmdr1*-edited parasites were identified by PCR and Sanger sequencing and cloned by limiting dilution. All primers used are listed in Table S6.

## Supplementary Material

adk9893_SupplementalMaterial

SupplementaryData1_SequencedSamples

SupplementaryData2_Compounds

SupplementaryData3_SNVs-INDELs

SupplementaryData4_CNVs

SupplementaryData5_CompoundTarget

SupplementaryData6_Pf6Genes

SupplementaryData7_Featured_SNVs

## Data Availability

The raw sequencing data generated in this study have been submitted to the NCBI Sequence Read Archive database (https://www.ncbi.nlm.nih.gov/sra/) under accession number PRJNA1022010. Previously published sequencing files were downloaded from the following SRA accession numbers: PRJNA385508, PRJNA504044, PRJNA560380, PRJNA299203, PRJNA253899, PRJNA226625, PRJNA308112, PRJNA315690, PRJNA167166, and SRP012591. All other data described in this study are in the Supplementary Material. Code created for this study is available at https://doi.org/10.5281/zenodo.13766513. Evolved lines that were created for this study are available from the originating lab as listed in Data S1.
