# Supplementary material for: Systematic in vitro evolution in Plasmodium falciparum reveals key determinants of drug resistance
Source: Science. Author manuscript; Available in PMC 2025 Feb 10. (PMC11809290; doi:10.1126/science.adk9893)
Supplement: adk9893_SupplementalMaterial [file NIHMS2048195-supplement-adk9893_SupplementalMaterial.pdf]

Science
AAAS

Supplementary Materials for

Systematic in vitro evolution in *Plasmodium falciparum* reveals key determinants of drug resistance

Madeline R. Luth†, Karla P. Godinez-Macias†, Daisy Chen†, John Okombo, Vandana Thathy, Xiu Cheng, Sindhu Daggupati, Heledd Davies, Satish K. Dhingra, Jan M. Economy, Rebecca C. S. Edgar, Maria G. Gomez-Lorenzo, Eva S. Istvan, Juan Carlos Jado, Gregory M. LaMonte, Bruno Melillo, Sachel Mok, Sunil K. Narwal, Tolla Ndiaye, Sabine Ottilie, Sara Palomo Diaz, Heekuk Park, Stella Peña, Frances Rocamora, Tomoyo Sakata-Kato, Jennifer L. Small-Saunders, Robert L. Summers, Patrick K. Tumwebaze, Manu Vanaerschot, Guoqin Xia, Tomas Yeo, Ashley You, Francisco-Javier Gamo, Daniel E. Goldberg, Marcus C.S. Lee, Case W. McNamara, Daouda Ndiaye, Philip J. Rosenthal, Stuart L. Schreiber, Gloria Serra, Jair Lage De Siqueira-Neto, Tina S. Skinner-Adams, Anne-Catrin Uhlemann, Nobutaka Kato, Amanda K. Lukens, Dyann F. Wirth, David A. Fidock, Elizabeth A. Winzeler

Correspondence to: ewinzeler@health.ucsd.edu

†These authors contributed equally to this work

The PDF file includes:

Figs. S1 to S12
Tables S1 to S10

Other Supplementary Materials for this manuscript include the following:

Data S1 to S7

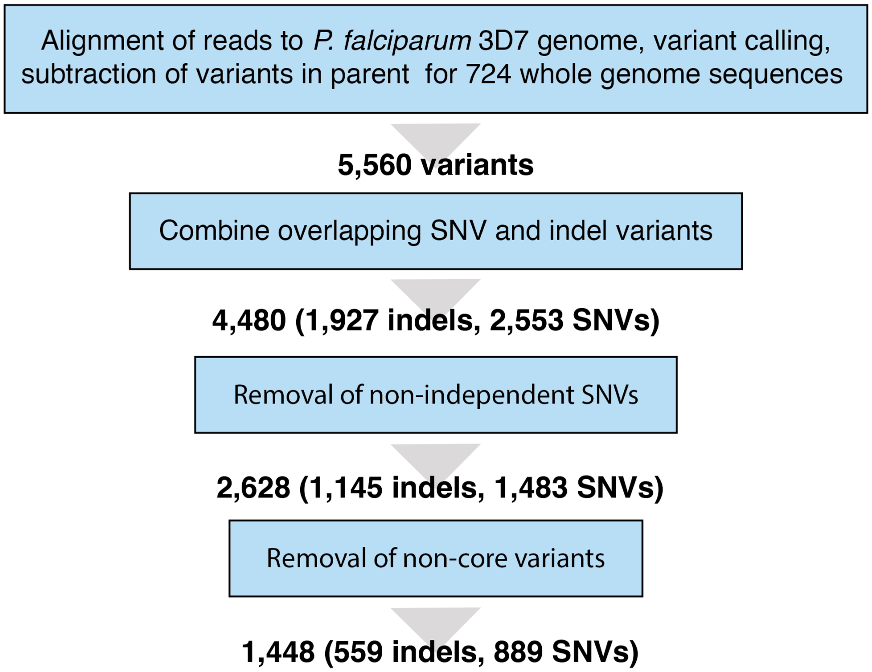

Fig. S1. Indels and SNV detection workflow.

Pipeline for variant detection and filtering to identify variants among the 724 clones analyzed. These variants were used as part of **Fig. 1**.

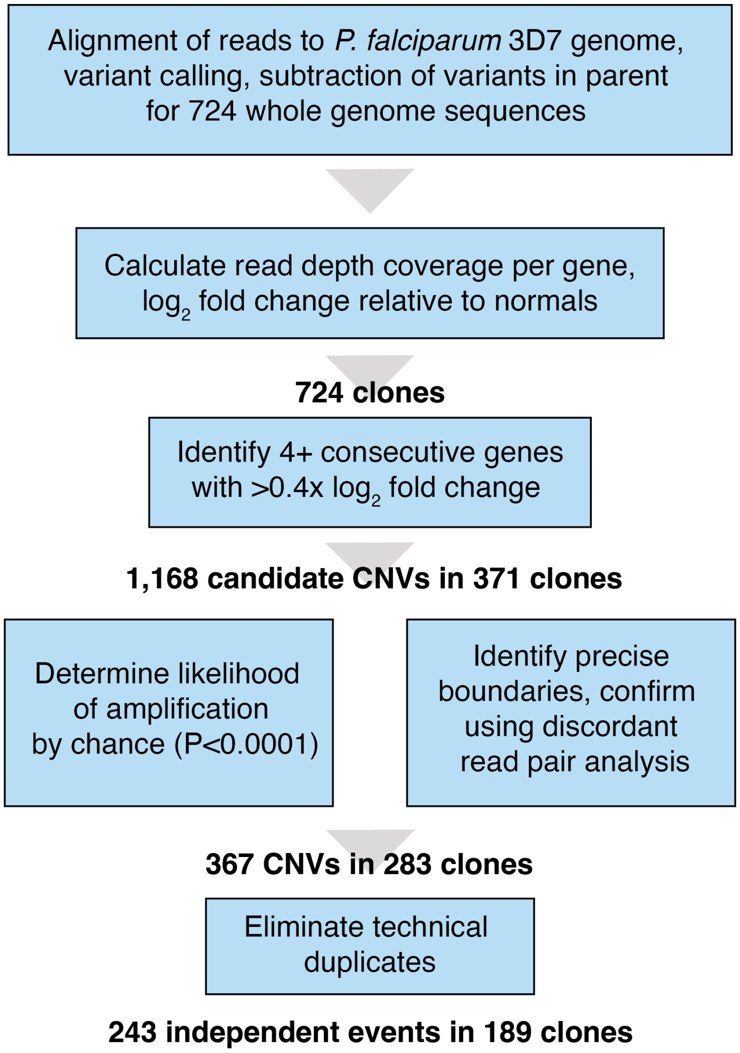

Fig. S2. CNV detection workflow.

Pipeline for CNV detection and filtering to identify amplification CNVs among the 724 clones analyzed. These CNVs were used as part of **Fig. 2**.

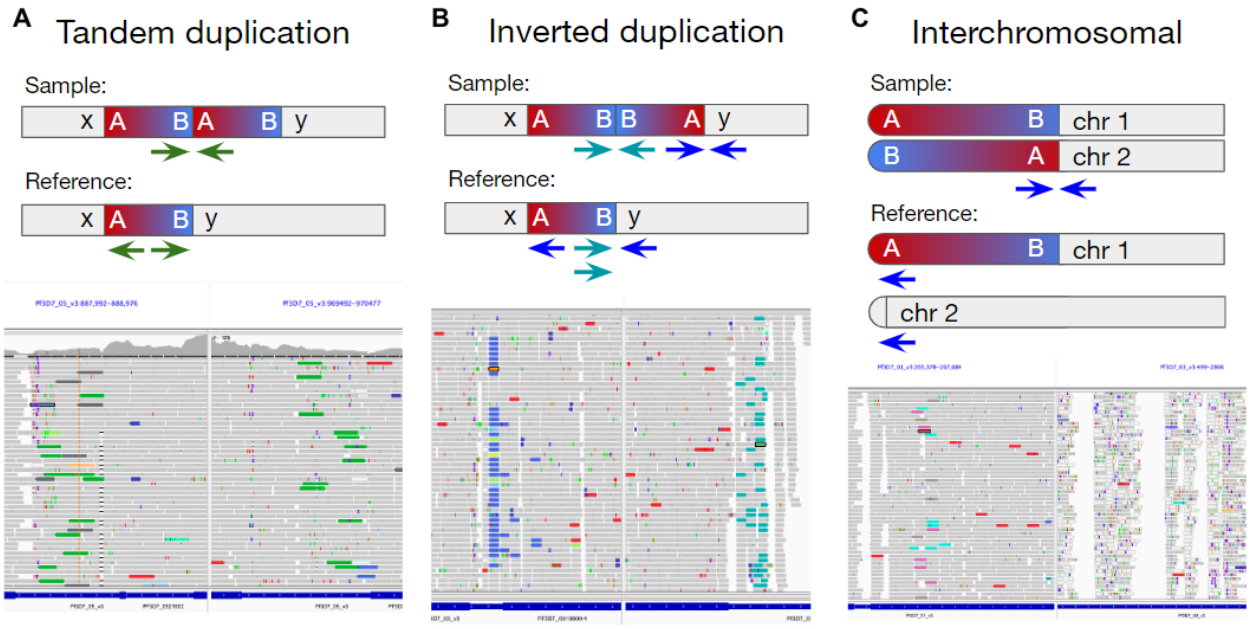

Fig. S3. Validation of CNVs near boundaries of structural events using discordant read pair evidence.

Manual inspection of example CNVs was performed with Integrative Genomics Viewer (IGV v#2.16 (*95*)). Raw paired end sequencing files for CNV-containing *P. falciparum* samples were loaded and displayed with default settings. Discordant read pair examples are shown for each structural variant type including **(A)** tandem duplication, **(B)** inverted duplication, and **(C)** interchromosomal rearrangements. The green (right-left, or RL), dark blue (RR) and teal (LL) read colors indicate non-normal read pair orientation that imply duplication or inversion with respect to the reference genome. The light blue and pink reads in **(C)** have non-normal insert size due to their mates mapping to different chromosomes.

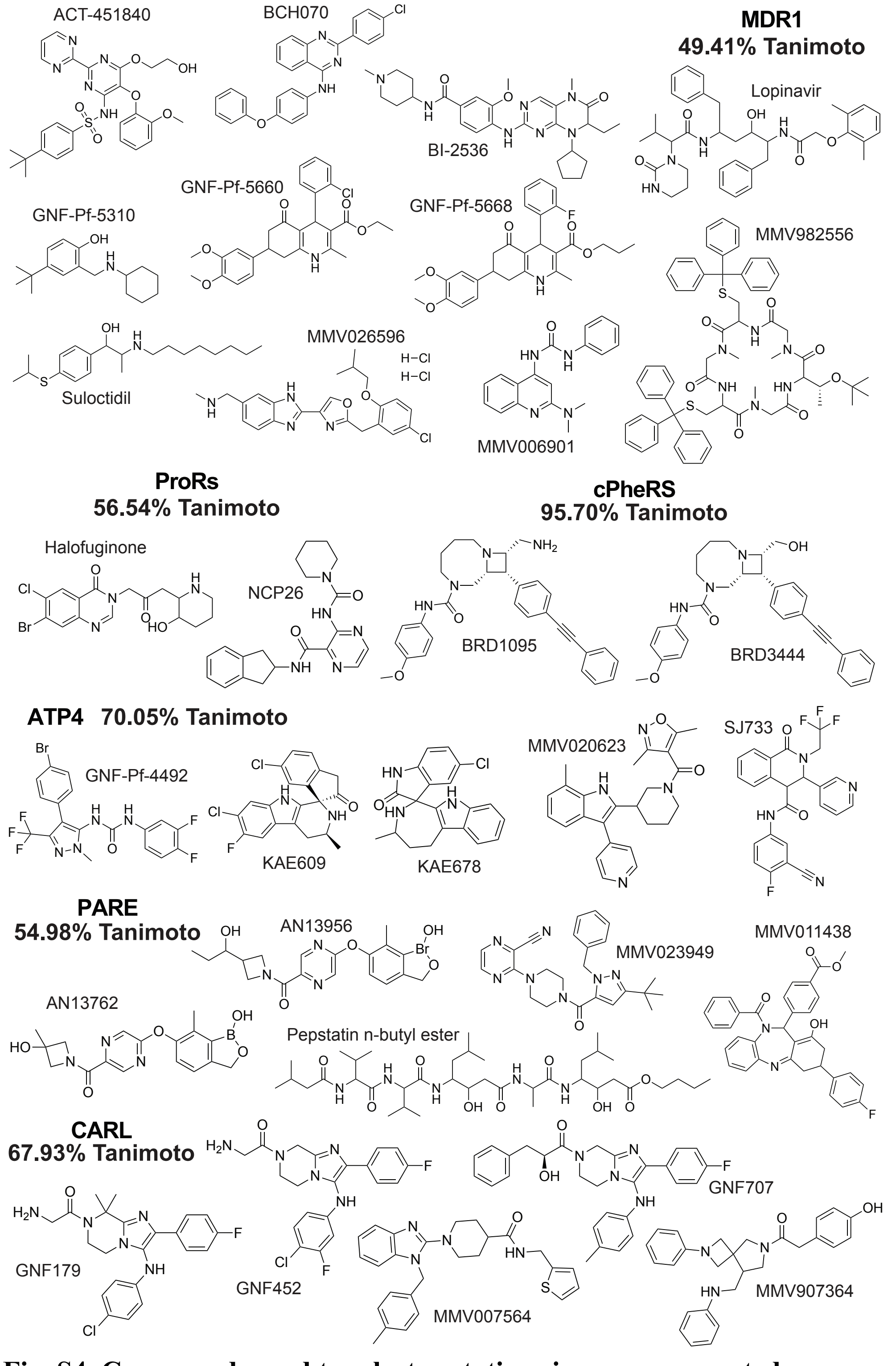

Fig. S4. Compounds used to select mutations in overrepresented genes.

Structures of compounds that gave rise to SNVs or indels in a subset of overrepresented genes (*pfmdr1*, *pfproRS*, *pfcpheRS*, *pfatp4*, *pfpare*, *pfcarl*) are shown. Compound SMILES (**Data S2)** were displayed using ChemDraw 22.2.0. Tanimoto similarity was calculated using RDKIT v. 2018.03.4.

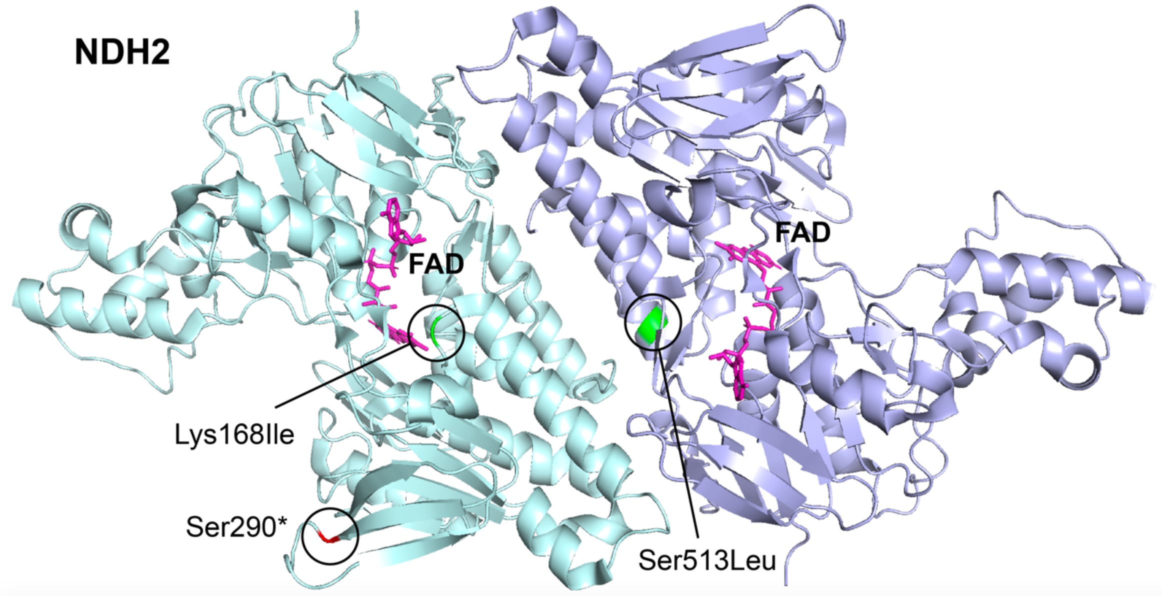

Fig. S5. Structural location of missense and nonsense mutations in PfNDH2 found in GNF-Pf-5611 resistant parasites.

Crystal structure of PfNDH2 (gene: PF3D7_0915000, annotated as type II NADH:ubiquinone oxidoreductase) homodimer was obtained from Yang et al. 2017 (*32*) (PDB ID: 5JWA). Modeling of three disruptive mutations in PfNDH2 found in parasites with evolved resistance to GNF-Pf-5611 using PyMOL Molecular Graphics System (Version 3.0; Schrödinger LLC) shows that the two missense mutations (K168I and S513L) are positioned near binding sites of ligand FAD, flavin adenine dinucleotide, while the nonsense mutation S290* is not.

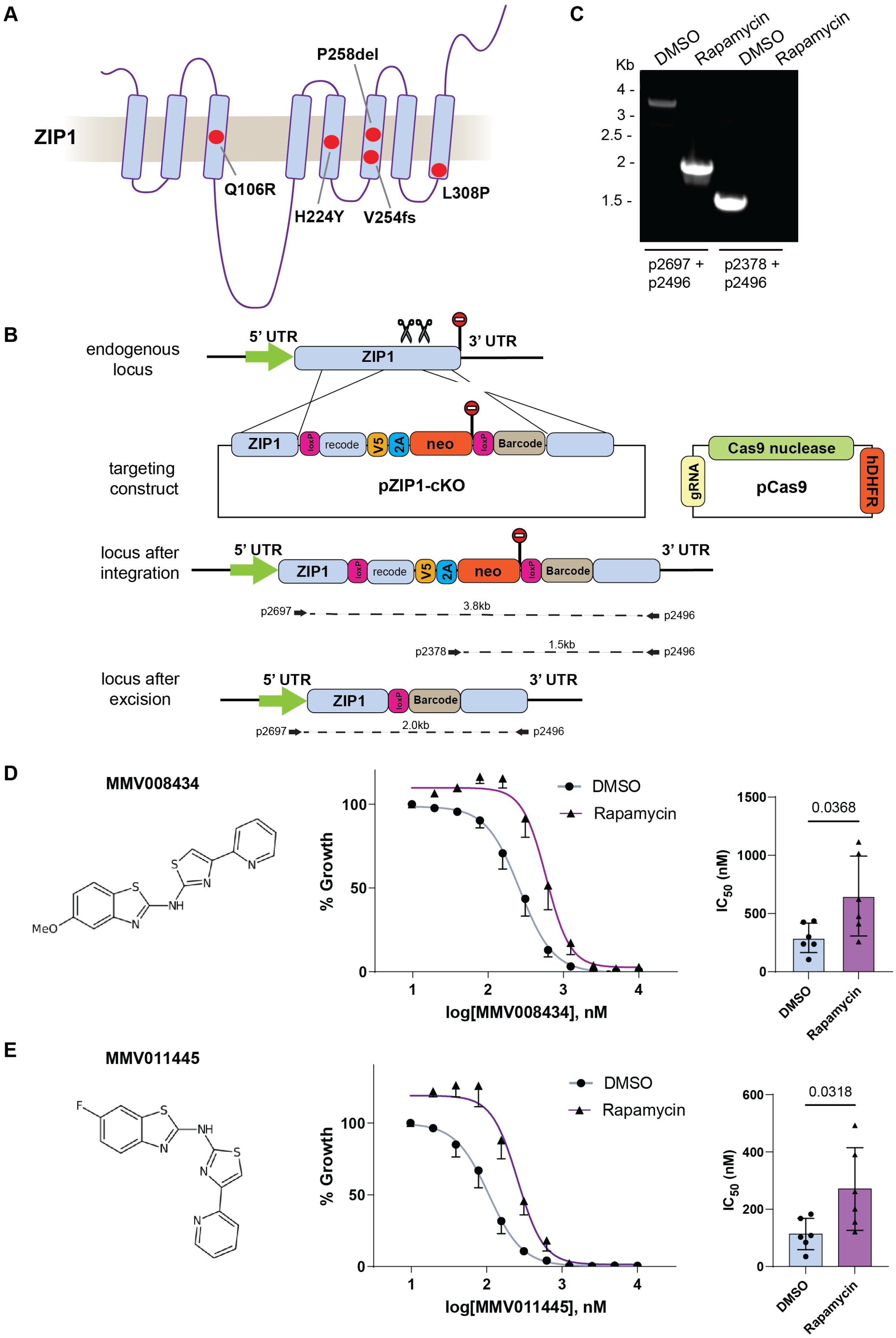
A
ZIP1
P258del
Q106R
H224Y
V254fs
L308P
C
Kb
DMSO
Rapamycin
DMSO
Rapamycin
4 -
3 -
2.5 -
2 -
1.5 -
p2697 + p2496
p2378 + p2496
B
endogenous locus
5' UTR
ZIP1
3' UTR
targeting construct
ZIP1
loxP
recode
V5
2A
neo
loxP
Barcode
pZIP1-cKO
Cas9 nuclease
gRNA
pCas9
hDHFR
locus after integration
5' UTR
ZIP1
loxP
recode
V5
2A
neo
loxP
Barcode
3' UTR
p2697
3.8kb
p2496
p2378
1.5kb
p2496
locus after excision
5' UTR
ZIP1
loxP
Barcode
3' UTR
p2697
2.0kb
p2496
D
MMV008434
MeO
S
N
NH
S
N
N
% Growth
100
50
0
1
2
3
4
log[MMV008434], nM
DMSO
Rapamycin
IC50 (nM)
1500
1000
500
0
0.0368
DMSO
Rapamycin
E
MMV011445
F
S
N
NH
S
N
N
% Growth
100
50
0
1
2
3
4
log[MMV011445], nM
DMSO
Rapamycin
IC50 (nM)
600
400
200
0
0.0318
DMSO
Rapamycin

Fig. S6. Conditional knockout of PfZIP1 confers resistance to MMV008434 and its analog MMV011445.

(**A**) Schematic of the protein structure of PfZIP1 (gene PF3D7_0609100, annotated as zinc transporter, putative) indicating predicted transmembrane domains and mutations identified in MMV008434 resistance selection. (**B**) Schematic of the *pfzip1* locus before and after editing with the indicated targeting construct. The pZIP1-cKO plasmid contained a 5' homology region followed by a synthetic intron containing the loxP excision site, with the remainder of the gene fused to a V5 epitope tag, a 2A skip peptide and a neomycin selection cassette. The second synthetic intron containing a loxP site followed this, as well as a genetic barcode. This targeting construct was co-transfected with a Cas9 plasmid containing a PfZIP1 specific guide, as well the selectable marker human dihydrofolate reductase (hDHFR). Arrows indicate oligonucleotides used in diagnostic PCRs as well as their expected sizes. (**C**) Diagnostic PCR of ZIP1-cKO parasites 24 h after treatment with DMSO or rapamycin resulting in excision at the loxP sites. PCR was performed using the oligonucleotide pairs outlined in (**B**). (**D-E**) Left panels: Chemical structure of MMV008434 (**D**) and MMV011445 (**E**). Right panels: Dose-response curves and IC_{50} values, as measured over a standard 72 h SYBR Green I assay. The IC_{50} values were calculated from six biological replicates performed in triplicate and data plotted as the mean ± standard error of the mean, with the inlay bar graphs showing the mean IC_{50} values of these replicates with statistical significance determined using an unpaired *t* test.

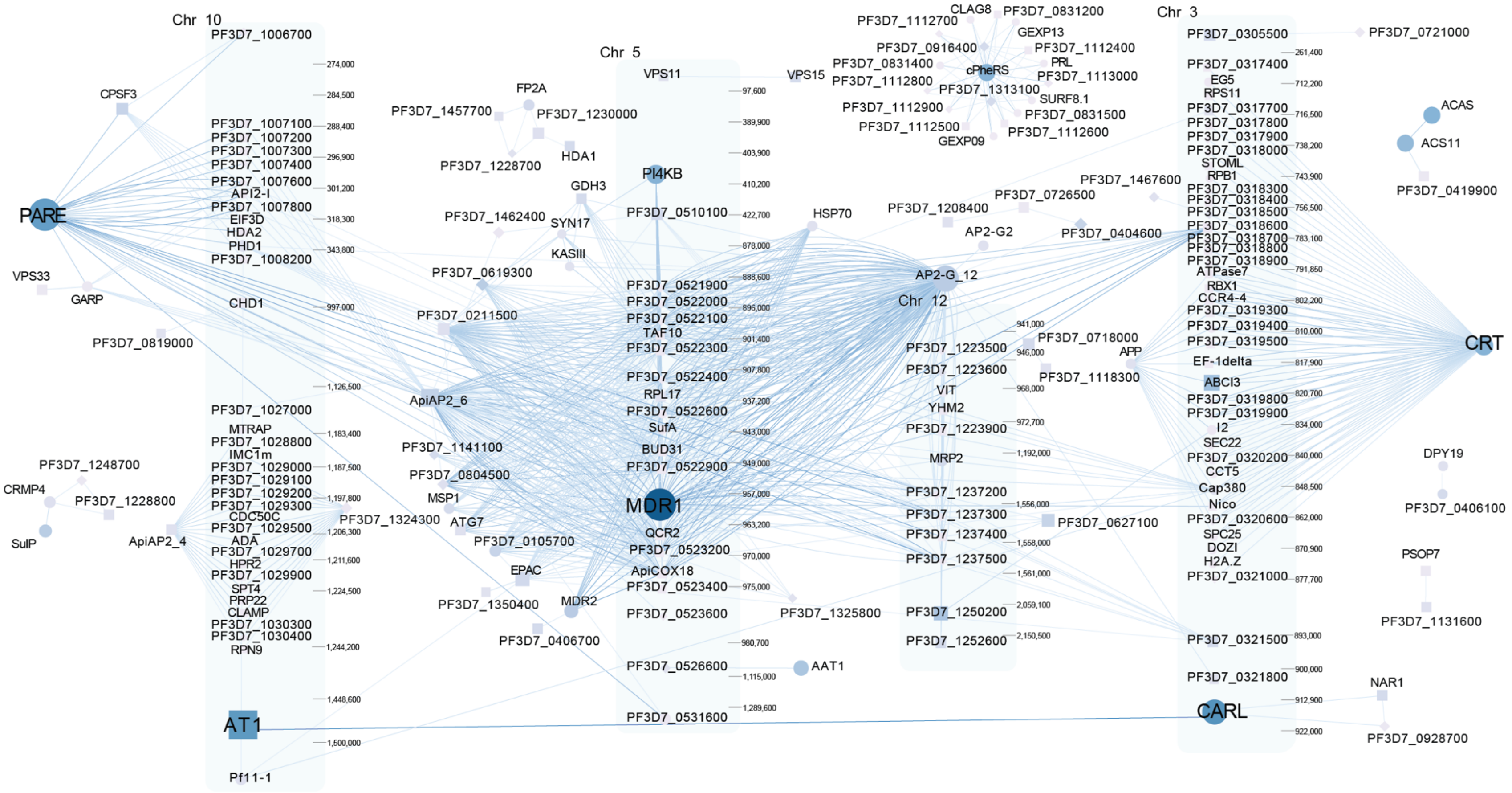

Fig. S7. Full gene interaction network based on shared mutations in selections with different compounds.

Schematic of the full gene-gene network linking two genes for which the same pair of mutations arose from selections with distinct compounds. Circles represent known proteins, diamonds conserved proteins, and squares putative proteins according to PlasmoDB version 63 Pf3D7 genome annotations. Node size is proportional to variant score (**Methods**), based on the number of overall mutations for each gene adjusting for SNV/indel occurrences. The strength of the color intensity represents the occurrence of disruptive mutation types from low (light blue) to high (dark blue) adjusting for non-missense mutations. Genes found in proximity to one another in the genome more frequently than expected were arranged spatially and highlighted (chromosomes 3, 5, 10, and 12).

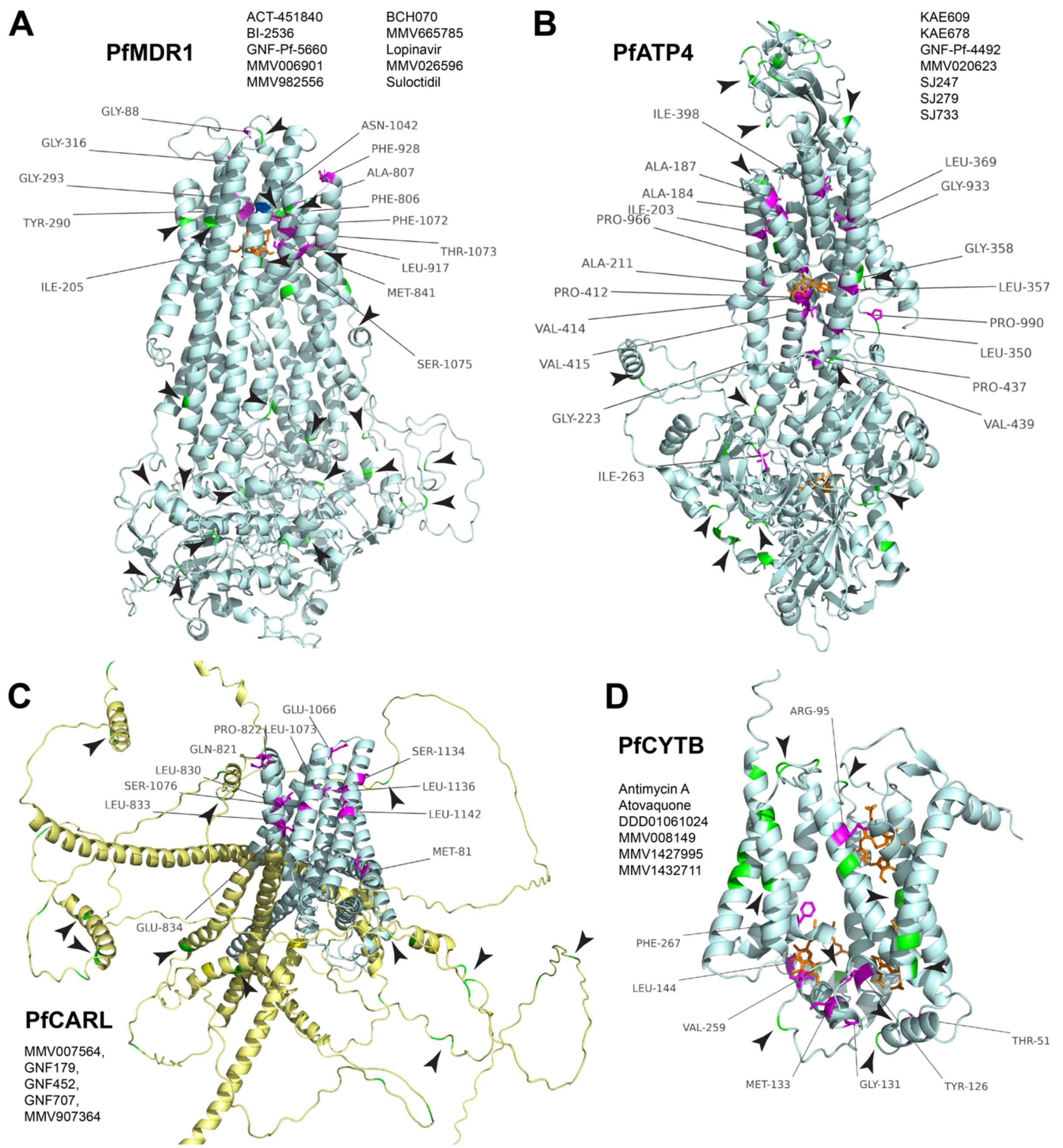

Fig. S8. Comparison of compound-selected vs. naturally occurring mutated residues in predicted structures of PfMDR1, PfATP4, PfCARL, and PfCYTB.

Models of **(B)** PfATP4 (AF-A0A143ZZK9-F1-model_v2), **(C)** PfCARL (AF-C0H483-F1-model_v4), and **(D)** PfCYTB (AFQ7HP03) were obtained from AlphaFold. **(A)** The PfMDR1 homology model was constructed with SWISS-MODEL (*43*) using 7a69 (Nanodisc reconstituted human ABCB1 in complex with MRK16 Fab and vincristine) as template. Ligands were added to AlphaFold models using AlphaFill (*96*) (PfCYTB: heme, stigmatellin; PfATP4: analog of cyclopiazonic acid with PDB ligand identifier CZA) or by merging structures in PyMOL (PfMDR1: vincristine). Regions with a high predicted aligned error (yellow) in AlphaFold models

for PfCARL were from residues 1 to 76, 157 to 782, 834 to 1,039. Amino acid residues that were mutated in our dataset are colored magenta. Amino acids residues with a field mutation and a global major allele frequency (gMAF) of greater than 0.002 were obtained from (*39*) and are colored green. Residues with variants that were found in both sets are colored blue. For PfCYTB, Thr-51 is a silent mutation. Field mutation sites are indicated by filled arrow heads (**Data S7**). The names of compounds that gave rise to mutations in the indicated protein through in vitro selections are listed.

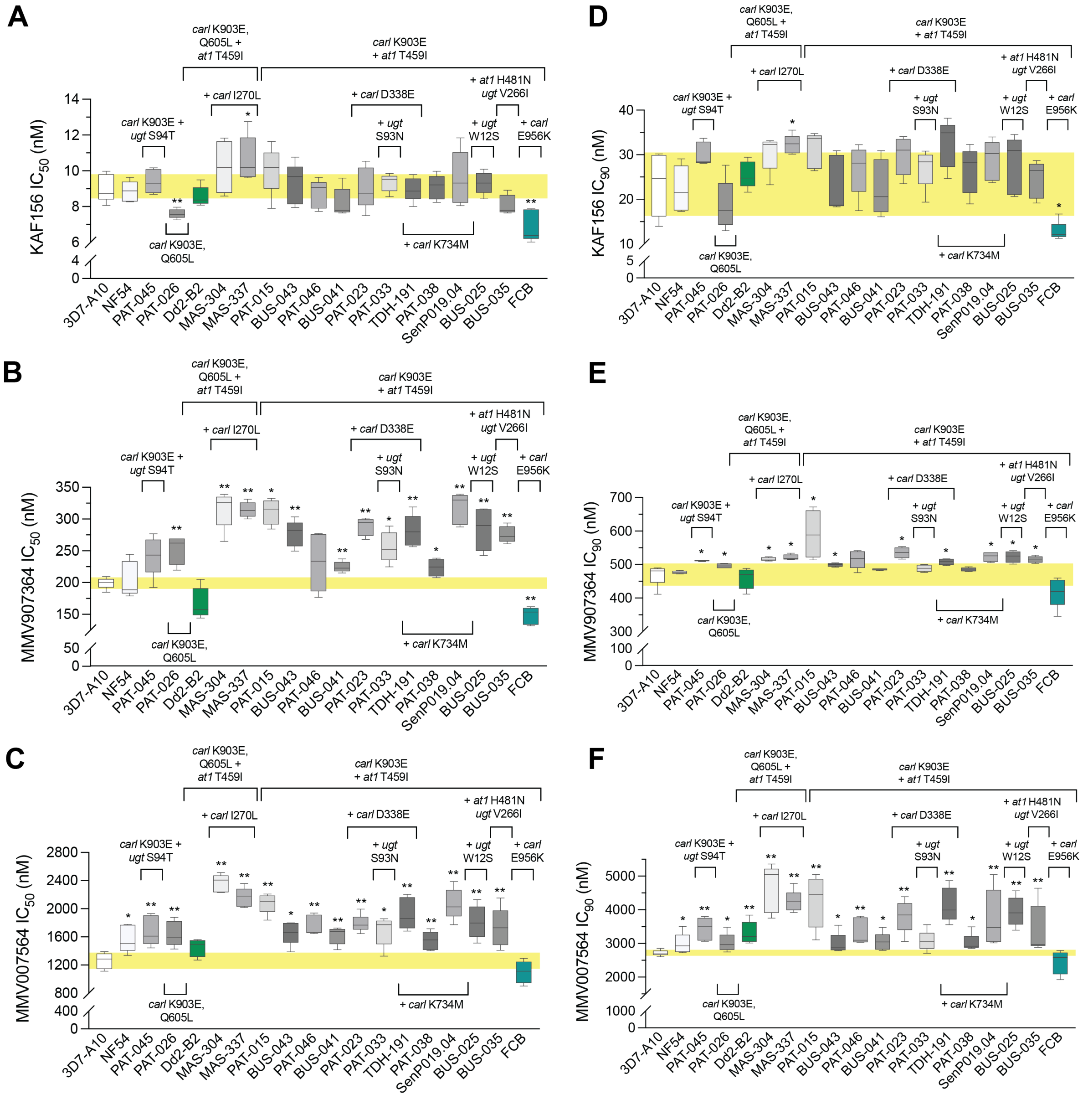

Fig. S9. In vitro 72 h activity of 3 compounds against 19 parasite lines with naturally occurring *pfcarl* missense mutations.

(**A-C**) IC_{50} and (**D-F**) IC_{90} data are presented as means ± SEMs (N, n = 4-5, 2) for each parasite line-compound pair. Lab lines and clinical isolates were manually hierarchically ordered and annotated to summarize missense variants in *pfcarl* (PF3D7_0321900), *pfat1* (PF3D7_1036800) and *pfugt* (PF3D7_1113300). Statistical significance was determined using Mann-Whitney U tests by comparing each line against the drug-sensitive wild-type control 3D7-A10; $^{**}P \leq 0.01$, $^{*}P \leq 0.05$. Mean IC_{50} or IC_{90} of 3D7-A10 ± 1 S.D. are represented by yellow bands. Mean IC_{50} and IC_{90} values for all compounds and lines are reported in **Table S4**.

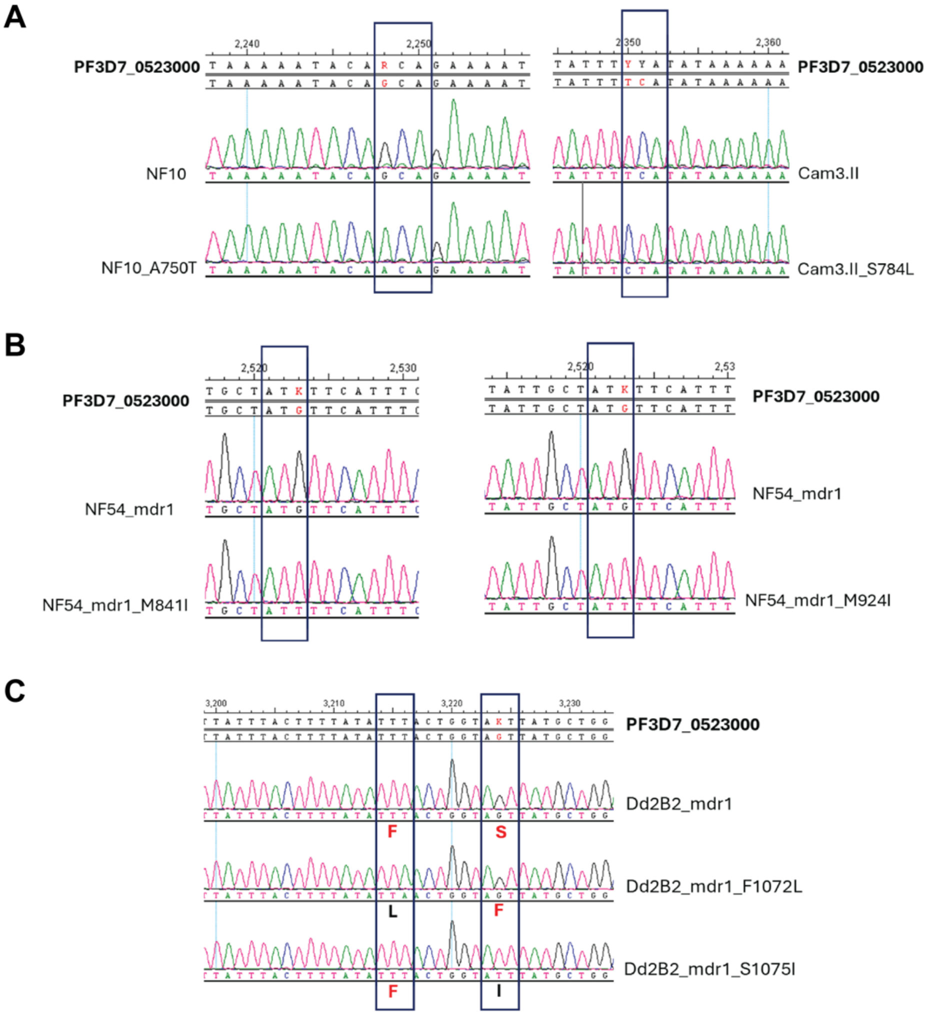

Fig. S10. Sanger sequencing confirms presence of edited mutations of interest in *pfmdr1*.
Sequence analysis confirms that missense mutations listed in **Table S5** are indeed present in the edited lines and absent in their isogenic parents. These lines were tested for susceptibility to compounds that select for PfMDR1 mutations.

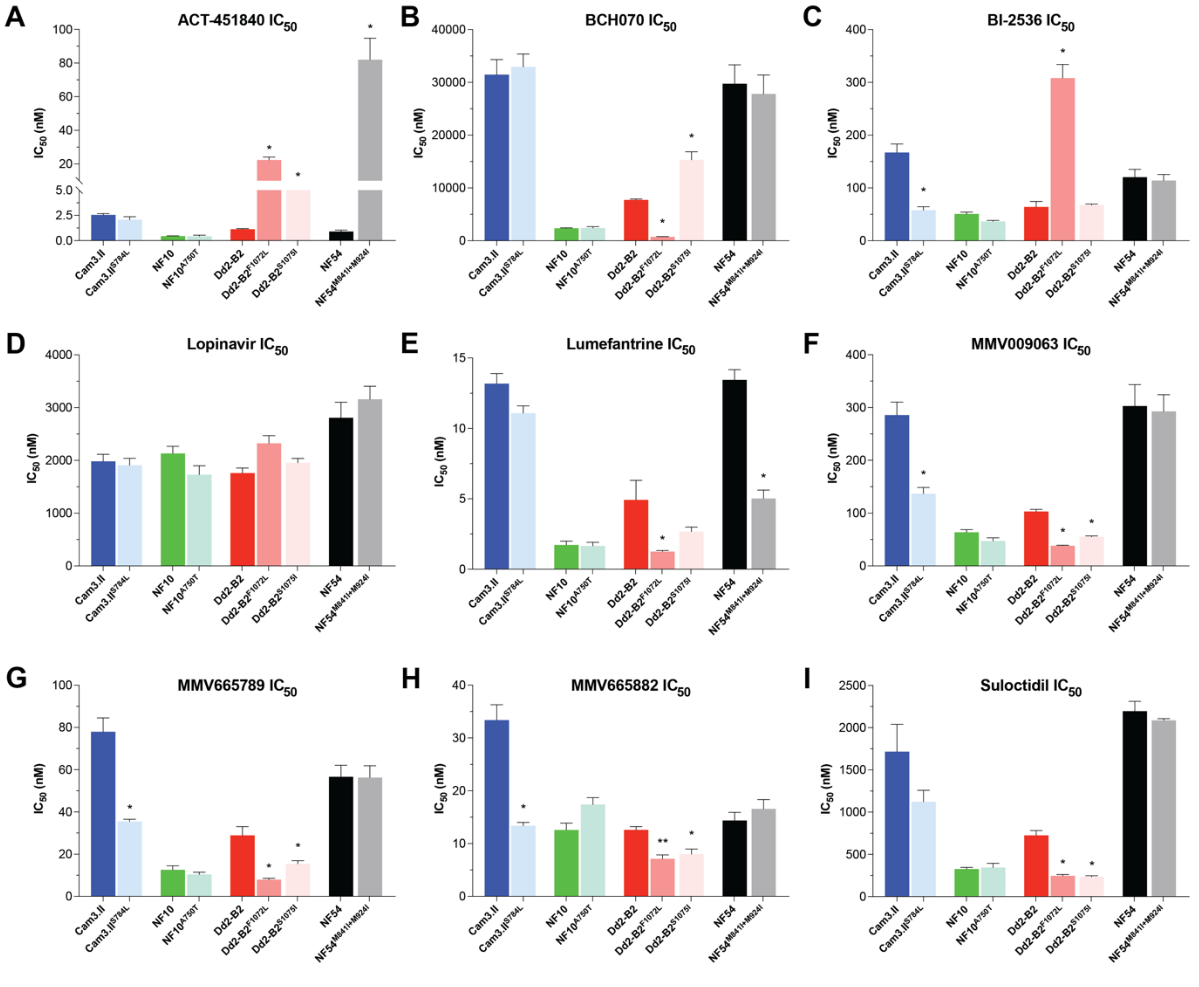

Fig. S11: In vitro 72 h activity of 9 compounds against PfMDR1 mutant ABS parasites.

IC_{50} data are presented as means ± SEMs (N, n = 4-5, 2) for each parasite line-compound pair. PfMDR1 mutations, originally found either in the field or from resistance selections, were edited into parental lines and verified using Sanger sequencing (**Fig. S10**). Statistical significance was determined using Mann-Whitney U tests by comparing each edited mutant line to its isogenic parent; $^{**}P \leq 0.01$, $^{*}P \leq 0.05$. Mean IC_{50} and IC_{90} values for all compounds and lines are reported in **Table S7**.

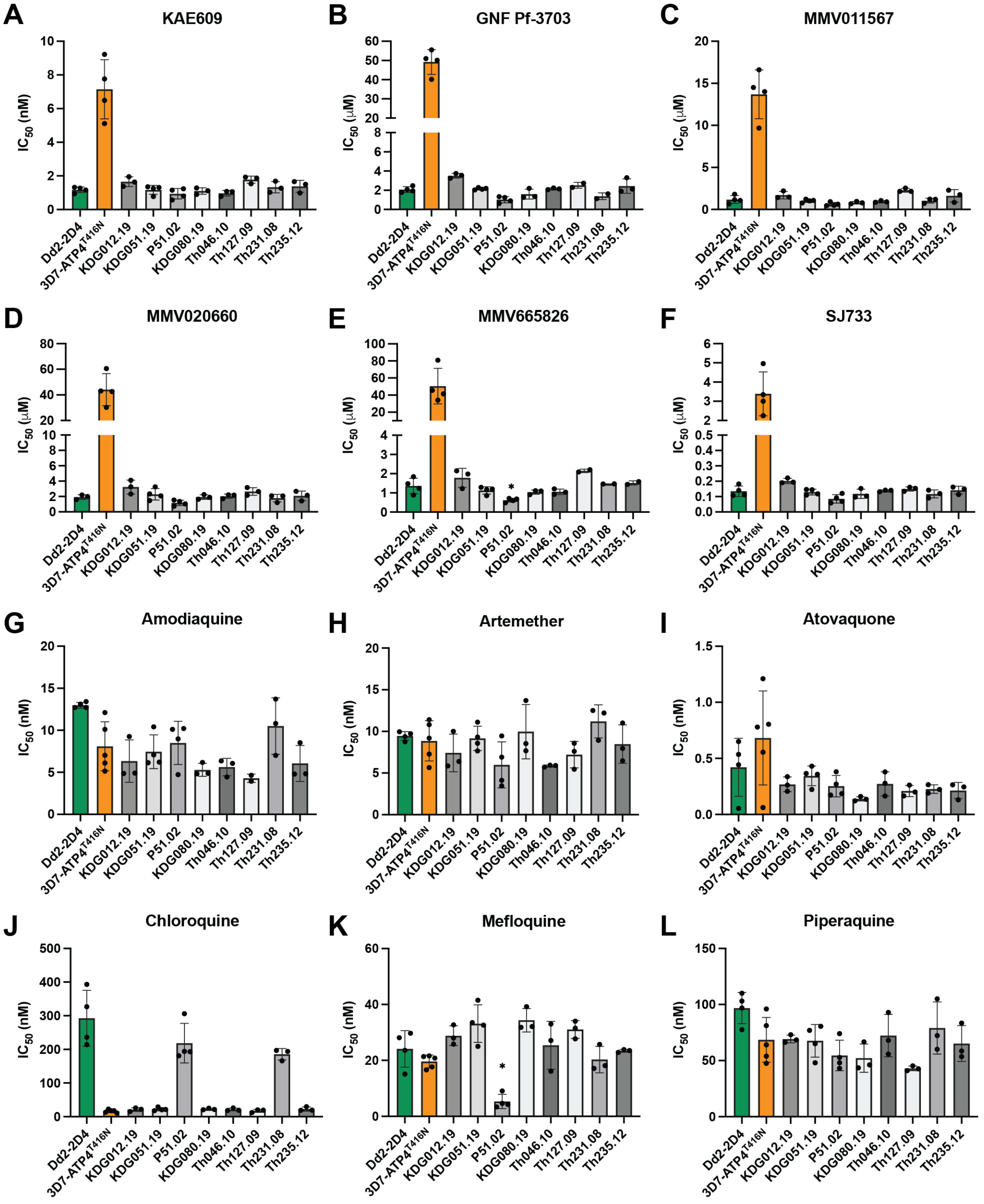

Fig. S12: In vitro 72 h activity of 12 compounds against 10 parasite lines with in vitro evolved or naturally occurring *pfatp4* missense mutations.

IC_{50} data are presented as means ± SEMs (N, *n* = 2-5, 3) for each parasite line-compound pair. All lines have in vitro evolved (3D7-ATP4^{T416N}) or naturally occurring variants in *pfatp4*, as confirmed by WGS (**Table S8**). Mean IC_{50} values for all compounds and lines are reported in **Table S10**.

Table S1. List of overrepresented genes in the compound-selected SNV/indel mutations dataset.

List of 128 genes mutated in more than one independent event (independent clone) in this study's dataset (**Data S3**). "Number of times mutated" refers to the number of independent clones the gene was mutated in, considering non-synonymous SNVs/indels only. "Number of unique compounds" counts the unique compounds used to select for the clones containing SNVs/indels in the gene. Probability of the observed number of occurrences was calculated in Excel using a hypergeometric null distribution assuming all Pf3D7 genes have equal chance of being mutated among all observed independent events in which a gene contained a non-synonymous SNV/indel. The negative $\log_{10}$ hypergeometric probability is also shown.

Gene ID	Gene Symbol	Gene Description	# of times mutated	# of unique compounds	Hypergeometric probability	Negative log probability
PF3D7_1211900	ATP4	non-SERCA-type Ca2+ -transporting P-ATPase	26	7	1.29E-53	52.89
PF3D7_0709700	PARE	prodrug activation and resistance esterase	20	5	3.21E-39	38.49
PF3D7_0523000	MDR1	multidrug resistance protein, MDR1	19	11	6.75E-37	36.17
PF3D7_1222600	AP2-G	AP2 domain transcription factor AP2-G	15	13	7.34E-28	27.13
PF3D7_0321900	CARL	cyclic amine resistance locus protein	14	5	1.13E-25	24.95
PF3D7_1036800	AT1	acetyl-CoA transporter, putative	13	2	1.62E-23	22.79
PF3D7_0509800	PI4KB	phosphatidylinositol 4-kinase	9	6	2.92E-15	14.54
PF3D7_0709000	CRT	chloroquine resistance transporter	9	6	2.92E-15	14.54
PF3D7_0109800	cPheRS	phenylalanine--tRNA ligase alpha subunit	8	2	2.62E-13	12.58
PF3D7_0627800	ACAS	acetyl-CoA synthetase, putative	8	3	2.62E-13	12.58
PF3D7_1238800	ACS11	acyl-CoA synthetase	8	4	2.62E-13	12.58
PF3D7_0312500	MFR3	major facilitator superfamily-related transporter, putative	7	2	2.09E-11	10.68
PF3D7_0603300	DHODH	dihydroorotate dehydrogenase	7	4	2.09E-11	10.68
PF3D7_1332900	cyto-IRS	isoleucine--tRNA ligase, putative	7	3	2.09E-11	10.68
PF3D7_0319700	ABCI3	ABC transporter I family member 1, putative	6	3	1.45E-09	8.84
PF3D7_1451100	eEF2	elongation factor 2	6	1	1.45E-09	8.84
PF3D7_0629500	AAT1	amino acid transporter AAT1	5	3	8.59E-08	7.07
PF3D7_1250200		CSC1-like protein, putative	5	2	8.59E-08	7.07
PF3D7_1255200	VAR	erythrocyte membrane protein 1, PfEMP1	5	5	8.59E-08	7.07
PF3D7_1417400		rap guanine nucleotide exchange factor, putative, pseudogene	5	5	8.59E-08	7.07
PF3D7_1447900	MDR2	multidrug resistance protein 2	5	5	8.59E-08	7.07
PF3D7_0404600		conserved Plasmodium membrane protein, unknown function	4	4	4.22E-06	5.37
PF3D7_0421300	VAR	erythrocyte membrane protein 1, PfEMP1	4	4	4.22E-06	5.37
PF3D7_0619300		conserved Plasmodium protein, unknown function	4	4	4.22E-06	5.37
PF3D7_0627100		ankyrin-repeat protein, putative	4	4	4.22E-06	5.37
PF3D7_0632500	VAR	erythrocyte membrane protein 1, PfEMP1	4	4	4.22E-06	5.37
PF3D7_0909700		FHA domain protein, putative	4	1	4.22E-06	5.37
PF3D7_0915000	NDH2	type II NADH:ubiquinone oxidoreductase	4	2	4.22E-06	5.37
PF3D7_1229100	MRP2	multidrug resistance-associated protein 2	4	3	4.22E-06	5.37
PF3D7_1240400	VAR	erythrocyte membrane protein 1, PfEMP1	4	4	4.22E-06	5.37
PF3D7_1471200	SulP	inorganic anion exchanger, inorganic anion antiporter	4	2	4.22E-06	5.37
PF3D7_0100100	VAR	erythrocyte membrane protein 1, PfEMP1	3	2	1.65E-04	3.78
PF3D7_0105700		asparagine-rich antigen Pfa35-2	3	3	1.65E-04	3.78
PF3D7_0417200	DHFR-TS	bifunctional dihydrofolate reductase-thymidylate synthase	3	1	1.65E-04	3.78
PF3D7_0418600		regulator of chromosome condensation, putative	3	2	1.65E-04	3.78
PF3D7_0510100		KH domain-containing protein, putative	3	3	1.65E-04	3.78
PF3D7_0609100	ZIP1	zinc transporter ZIP1, putative	3	1	1.65E-04	3.78
PF3D7_0613800	ApiAP2	AP2 domain transcription factor, putative	3	3	1.65E-04	3.78
PF3D7_0732700	RIF	rifin	3	3	1.65E-04	3.78
PF3D7_1008100	PHD1	conserved Plasmodium protein, unknown function	3	3	1.65E-04	3.78
PF3D7_1115700	FP2A	cysteine proteinase falcipain 2a	3	3	1.65E-04	3.78
PF3D7_1116700	DPAP1	dipeptidyl aminopeptidase 1	3	1	1.65E-04	3.78
PF3D7_1147500	FTB	protein farnesyltransferase subunit beta	3	1	1.65E-04	3.78
PF3D7_1205500		zinc finger protein, putative	3	3	1.65E-04	3.78
PF3D7_1213800	PRS	proline--tRNA ligase	3	2	1.65E-04	3.78
PF3D7_1230000		TBC domain-containing protein, putative	3	3	1.65E-04	3.78
PF3D7_1328100		proteasome subunit beta type-7, putative	3	1	1.65E-04	3.78
PF3D7_1359900		conserved Plasmodium membrane protein, unknown function	3	2	1.65E-04	3.78
PF3D7_1366300		conserved Plasmodium protein, unknown function	3	3	1.65E-04	3.78
PF3D7_1427100		lipase, putative	3	3	1.65E-04	3.78
PF3D7_1438500	CPSF3	cleavage and polyadenylation specificity factor subunit 3, putative	3	2	1.65E-04	3.78
PF3D7_1462400		conserved Plasmodium protein, unknown function	3	3	1.65E-04	3.78
PF3D7_1464500		conserved Plasmodium membrane protein, unknown function	3	3	1.65E-04	3.78
mal_mito_2		cytochrome c oxidase subunit I (cox1)	2	2	4.82E-03	2.32
PF3D7_0103100	VPS51	vacuolar protein sorting-associated protein 51, putative	2	2	4.82E-03	2.32
PF3D7_0103200	NT4	nucleoside transporter 4	2	1	4.82E-03	2.32
PF3D7_0113000	GARP	glutamic acid-rich protein GARP	2	2	4.82E-03	2.32
PF3D7_0115700	VAR	erythrocyte membrane protein 1, PfEMP1	2	2	4.82E-03	2.32
PF3D7_0215500		conserved Plasmodium protein, unknown function	2	2	4.82E-03	2.32
PF3D7_0223200	RIF	rifin	2	2	4.82E-03	2.32

PF3D7_0305500		conserved Plasmodium protein, unknown function	2	2	4.82E-03	2.32
PF3D7_0321500		peptidase, putative	2	2	4.82E-03	2.32
PF3D7_0406100		V-type proton ATPase subunit B	2	2	4.82E-03	2.32
PF3D7_0406500		NYN domain-containing protein, putative	2	2	4.82E-03	2.32
PF3D7_0406700		conserved Plasmodium protein, unknown function	2	2	4.82E-03	2.32
PF3D7_0418000		conserved Plasmodium protein, unknown function	2	2	4.82E-03	2.32
PF3D7_0419900		phosphatidylinositol 4-kinase, putative	2	2	4.82E-03	2.32
PF3D7_0420300	ApiAP2	AP2 domain transcription factor, putative	2	2	4.82E-03	2.32
PF3D7_0511500		RNA pseudouridylate synthase, putative	2	2	4.82E-03	2.32
PF3D7_0606500	PTB	polypyrimidine tract-binding protein, putative	2	2	4.82E-03	2.32
PF3D7_0609500		conserved Plasmodium protein, unknown function	2	2	4.82E-03	2.32
PF3D7_0612200	LRR6	leucine-rich repeat protein	2	2	4.82E-03	2.32
PF3D7_0614200	NAR1	cytosolic Fe-S cluster assembly factor NAR1, putative	2	2	4.82E-03	2.32
PF3D7_0629900		sec14-like cytosolic factor or phosphatidylinositol/phosphatidylcholine transfer protein, putative	2	2	4.82E-03	2.32
PF3D7_0632800	VAR	erythrocyte membrane protein 1, PfEMP1	2	2	4.82E-03	2.32
PF3D7_0700100	VAR	erythrocyte membrane protein 1, PfEMP1	2	2	4.82E-03	2.32
PF3D7_0703900		conserved Plasmodium membrane protein, unknown function	2	2	4.82E-03	2.32
PF3D7_0718000		dynein heavy chain, putative	2	2	4.82E-03	2.32
PF3D7_0718300	CRMP2	cysteine repeat modular protein 2	2	2	4.82E-03	2.32
PF3D7_0721000		conserved Plasmodium membrane protein, unknown function	2	2	4.82E-03	2.32
PF3D7_0725300		conserved protein, unknown function	2	1	4.82E-03	2.32
PF3D7_0727100		conserved protein, unknown function	2	2	4.82E-03	2.32
PF3D7_0802000	GDH3	glutamate dehydrogenase, putative	2	2	4.82E-03	2.32
PF3D7_0806200	DPY19	Dpy-19-like C-mannosyltransferase, putative	2	2	4.82E-03	2.32
PF3D7_0818900	HSP70	heat shock protein 70	2	2	4.82E-03	2.32
PF3D7_0823000	VPS15	serine/threonine protein kinase VPS15, putative	2	2	4.82E-03	2.32
PF3D7_0828100		conserved Plasmodium protein, unknown function	2	2	4.82E-03	2.32
PF3D7_0833500	VAR	erythrocyte membrane protein 1, PfEMP1	2	2	4.82E-03	2.32
PF3D7_0908800		mitochondrial carrier protein, putative	2	1	4.82E-03	2.32
PF3D7_0914700	MFR4	major facilitator superfamily-related transporter, putative	2	1	4.82E-03	2.32
PF3D7_0915400	PFK9	ATP-dependent 6-phosphofructokinase	2	1	4.82E-03	2.32
PF3D7_0916400		conserved Plasmodium protein, unknown function	2	2	4.82E-03	2.32
PF3D7_0930300	MSP1	merozoite surface protein 1	2	2	4.82E-03	2.32
PF3D7_0935200	VPS33	vacuolar protein sorting-associated protein 33, putative	2	2	4.82E-03	2.32
PF3D7_0935400	GDV1	gametocyte development protein 1	2	2	4.82E-03	2.32
PF3D7_1008700		tubulin beta chain	2	1	4.82E-03	2.32
PF3D7_1011400		proteasome subunit beta type-5	2	1	4.82E-03	2.32
PF3D7_1027000		conserved Plasmodium protein, unknown function	2	2	4.82E-03	2.32
PF3D7_1038400	Pf11-1	gametocyte-specific protein (Pf11-1)	2	2	4.82E-03	2.32
PF3D7_1041300	VAR	erythrocyte membrane protein 1, PfEMP1	2	2	4.82E-03	2.32
PF3D7_1118300		insulinase, putative	2	2	4.82E-03	2.32
PF3D7_1126100	ATG7	autophagy-related protein 7, putative	2	2	4.82E-03	2.32
PF3D7_1128400	FPPS/ GGPPS	bifunctional farnesyl/geranylgeranyl diphosphate synthase	2	1	4.82E-03	2.32
PF3D7_1131400		conserved Plasmodium protein, unknown function	2	1	4.82E-03	2.32
PF3D7_1131600		kelch domain-containing protein, putative	2	2	4.82E-03	2.32
PF3D7_1141100		conserved Plasmodium protein, unknown function	2	2	4.82E-03	2.32
PF3D7_1208400		amino acid transporter, putative	2	2	4.82E-03	2.32
PF3D7_1219300	VAR	erythrocyte membrane protein 1, PfEMP1	2	2	4.82E-03	2.32
PF3D7_1248700		conserved Plasmodium protein, unknown function	2	2	4.82E-03	2.32
PF3D7_1252600		esterase, putative	2	2	4.82E-03	2.32
PF3D7_1300300	VAR	erythrocyte membrane protein 1, PfEMP1	2	2	4.82E-03	2.32
PF3D7_1311900	vapA	V-type proton ATPase catalytic subunit A	2	1	4.82E-03	2.32
PF3D7_1313100		conserved Plasmodium protein, unknown function	2	2	4.82E-03	2.32
PF3D7_1323800	VPS52	vacuolar protein sorting-associated protein 52, putative	2	2	4.82E-03	2.32
PF3D7_1345400		conserved Plasmodium protein, unknown function	2	2	4.82E-03	2.32
PF3D7_1355700	NIF3	NLI interacting factor-like phosphatase, putative	2	2	4.82E-03	2.32
PF3D7_1405400	MutS	DNA mismatch repair protein, putative	2	1	4.82E-03	2.32
PF3D7_1408200	AP2-G2	AP2 domain transcription factor AP2-G2, putative	2	2	4.82E-03	2.32
PF3D7_1409600		conserved Plasmodium protein, unknown function	2	2	4.82E-03	2.32
PF3D7_1411000.1		conserved Plasmodium protein, unknown function	2	2	4.82E-03	2.32
PF3D7_1412400		conserved Plasmodium protein, unknown function	2	2	4.82E-03	2.32
PF3D7_1417600		conserved Plasmodium protein, unknown function	2	2	4.82E-03	2.32
PF3D7_1433400	PHD2	zinc finger protein, putative	2	2	4.82E-03	2.32
PF3D7_1454400	APP	aminopeptidase P	2	2	4.82E-03	2.32
PF3D7_1459200		WD repeat-containing protein, putative	2	2	4.82E-03	2.32
PF3D7_1465800		dynein beta chain, putative	2	2	4.82E-03	2.32
PF3D7_1470100		conserved Plasmodium protein, unknown function	2	2	4.82E-03	2.32
PF3D7_1472200	HDA1	histone deacetylase, putative	2	2	4.82E-03	2.32

Table S2. Allele frequency of missense SNVs in *pfcarl*, *pfat1* and *pfugt* of laboratory lines and field isolates tested in dose-response assays.

	Frequencies of naturally occurring *pfcarl*, *pfat1*, and *pfugt* SNVs per isolate tested in dose-response assays[a]													
**Gene description**	*pfcarl* (cyclic amine resistance locus protein)							*pfat1* (acetyl-CoA transporter)		*pfugt* (UDP-galactose transporter)				
**Gene ID**	PF3D7_0321900							PF3D7_1036800		PF3D7_1113300				
**Exon ID**	2	2	2	2	2	2	2	5	5	1	1	1	1	
**Codon change**	Att/Ctt	gaT/gaG	cAg/cTg	atG/atA	aAg/aTg	Aaa/Gaa	Gaa/Aaa	Cat/Aat	aCa/aTa	tGg/tCg	aGt/aAt	aGt/aCt	Gta/Ata	
**Amino acid change**	I270L	D338E	Q605L	M668I	K734M	K903E	E956K	H481N	T459I	W12S	S93N	S94T	V266I	
**Pf6 AAF[b]**	0.039	0.104	0.249	0.015	0.072	0.998	0.008	0.048	0.916	0.002	0.006	0.000	0.002	
**Pf6 sample count[b]**	112	334	1171	52	259	4694	12	93	4056	6	11	0	5	**Geographic origin of isolate (Year of collection)[f]**
BUS-025	0	0	0	0	0	1	0	0	1	1	0	0	0	Eastern Uganda/EAF (2021)
BUS-035	0	0	0	0	0	1	0	1	1	0	0	0	1	Eastern Uganda/EAF (2021)
BUS-041	0	0.21	0	0	0	1	0	0	1	0	0	0	0	Eastern Uganda/EAF (2021)
BUS-043	0	0	0	0	0	1	0	0	1	0	0	0	0	Eastern Uganda/EAF (2021)
MAS-304	0.96	0	1	0	0	1	0	0	1	0	0	0	0	Eastern Uganda/EAF (2019)
MAS-337	0.83	0	0.91	0.17	0	1	0	0	1	0	0	0	0	Eastern Uganda/EAF (2019)
PAT-015	0	0	0	0	0	0.96	0	0	1	0	0	0	0	Northern Uganda/EAF (2021)
PAT-023	0	1	0	0	0	1	0	0	1	0	0	0	0	Northern Uganda/EAF (2021)
PAT-026	0	0	1	0	0	1	0	0	0	0	0	0	0	Northern Uganda/EAF (2021)
PAT-033	0	1	0	0	0	1	0	0	0.49	0	1	0	0	Northern Uganda/EAF (2021)
PAT-038	0	0	0	0	0.94	1	0	0	1	0	0	0	0	Northern Uganda/EAF (2021)
PAT-045	0	0	0	0	0	1	0	0	0	0	0	1	0	Northern Uganda/EAF (2021)
PAT-046	0	0.17	0	0	0	1	0	0	0.87	0	0	0	0	Northern Uganda/EAF (2021)
TDH-191	0	1	0	0	1	1	0	0	1	0	0	0	0	Eastern Uganda/EAF (2021)
SenP019.04	0	0	0	0	1	1	0	0	1	0	0	0	0	Pikine, Senegal/WAF (2004)
3D7-A10[c]	0	0	0	0	0	0	0	0	0	0	0	0	0	Rwanda/CAF (1979)[c,d]
NF54 (JHU)[d]	0	0	0	0	0	0	0	0	0	0	0	0	0	
Dd2-B2[e]	0	0	1	0	0	1	0	0	1	0	0	0	0	Thailand/SE Asia (1980)
FCB	0	0	0	0	0	1	1	0	1	0	0	0	0	SE Asia

[a] The in vitro susceptibilities of a set of 19 laboratory lines and culture-adapted field isolates to KAF156, MMV007564, and MMV907364 were measured using 72 h dose-response assays. Whole-genome sequencing was performed in-house using 300 bp paired-end dual index Illumina libraries prepared from freshly extracted parasite genomic DNA at the end of the assay period (see **Methods**). The *P. falciparum* 3D7 reference genome (PlasmoDB, v48.0) was used to identify naturally occurring single nucleotide variants (SNVs) in the tested strains (see **Methods**). Alternate allele frequencies of the discovered SNVs were derived from *observed alternate reads / sum of alternate and reference reads*.

[b] AAF, global alternate allele frequency in the Pf6 dataset (*39*) as described in **Data S7**, and number of samples from the full Pf6 dataset (n = 7,113) that have total depth ≥ 50 at the site and alternate allele frequency ≥ 0.5.

[c] 3D7-A10 is a sub-clone of 3D7, which was derived from NF54 (*97*).

[d] NF54 is a strain of African origin isolated in the Netherlands (*98*). NF54 (JHU) is a gametocyte-producing stock of NF54 obtained from Dr. Photini Sinnis, Johns Hopkins University, Baltimore, MD, USA.

[e] Dd2-B2 was cloned from Dd2, which is a multi-drug resistant line originating from W2, an Indochina III/CDC isolates (*80*).

[f] SenP019.04 (*99*) and the Ugandan field isolates have been previously described (*86*). EAF, East Africa; WAF, West Africa; CAF, Central Africa; SE Asia, Southeast Asia; year of collection was included if known.

Table S3. Whole-genome sequencing metrics for *pfcarl*, *pfat1* and *pfugt* SNV profiling of the 19 lines tested for susceptibility to compounds that give rise to PfCARL mutations.

Sample names		BUS-025	BUS-035	BUS-041	BUS-043	MAS-304	MAS-337	P019-04	PAT-015	PAT-023	PAT-026
Total reads		3,661,654	3,792,363	2,473,013	3,105,212	2,855,795	2,412,455	3,035,204	3,143,745	4,767,578	3,523,865
# Mapped reads		3,278,878	3,399,235	2,237,974	2,819,471	2,545,832	2,183,638	2,728,781	2,838,762	4,339,714	3,160,421
Duplication rate		25.58%	26.96%	25.68%	26.63%	27.96%	22.15%	29.57%	28.49%	39.22%	27.22%
General error rate		1.74%	1.63%	1.62%	1.59%	1.58%	1.65%	1.60%	1.55%	1.51%	1.61%
Mean mapping quality (Phred)		56.59	56.53	56.66	56.61	56.71	56.6	56.71	56.72	56.94	56.68
Depth of coverage	mean	30.94	32.33	20.88	26.83	23.86	20.89	25.32	26.68	39.56	29.79
	SD	25.34	24.86	19.3	22.22	22.14	18.07	20.5	22.02	32.13	24.41
% of PF genome with > x no. reads	1X	97.04%	96.63%	97.00%	96.38%	96.56%	96.93%	96.66%	96.05%	96.40%	96.72%
	5X	94.47%	93.75%	89.64%	92.36%	90.20%	91.81%	93.22%	91.65%	94.45%	93.34%
	10X	89.83%	87.80%	75.98%	84.19%	78.76%	81.14%	86.95%	83.03%	91.09%	86.82%
	20X	74.41%	71.16%	49.61%	64.64%	56.51%	52.81%	66.58%	62.63%	79.80%	69.50%
	30X	53.62%	53.71%	24.73%	42.69%	34.18%	21.72%	36.93%	41.97%	65.41%	50.10%

Sample names		PAT-033	PAT-038	PAT-045	PAT-046	TDH-191	3D7-A10	NF54-JHU	FCB	Dd2-B2
Total reads		4,556,377	2,873,575	3,439,036	3,353,816	3,637,172	3,259,532	3,394,001	3,479,009	3,003,549
# Mapped reads		4,103,027	2,584,204	3,132,973	2,816,801	3,228,807	2,996,321	3,007,200	3,043,756	2,701,350
Duplication rate		32.35%	25.66%	30.39%	33.64%	25.50%	22.88%	30.93%	25.65%	27.88%
General error rate		1.62%	1.63%	1.58%	1.74%	1.59%	0.74%	0.72%	1.78%	1.62%
Mean mapping quality (Phred)		56.75	56.59	56.78	56.58	56.52	58.82	59.16	56.4	56.65
Depth of coverage	mean	38.11	24.35	29.32	24.75	30.82	29.52	27.09	28.11	25.38
	SD	31.08	21.68	25.28	35.32	30.4	25.28	28.57	29.07	24.76
% of PF genome with > x no. reads	1X	97.54%	97.30%	96.73%	94.20%	96.62%	97.33%	99.13%	96.20%	95.71%
	5X	95.40%	91.69%	93.37%	78.68%	92.35%	95.87%	97.74%	93.80%	92.81%
	10X	91.27%	81.03%	86.53%	65.67%	84.98%	91.21%	93.15%	89.47%	87.05%
	20X	78.16%	58.54%	68.39%	48.19%	68.08%	73.39%	72.65%	72.05%	66.43%
	30X	63.58%	34.88%	48.54%	32.89%	51.77%	49.27%	40.71%	43.66%	36.11%

Table S4. Dose-response data for lines with naturally occurring PfCARL mutations. The in vitro susceptibilities of a collection of four laboratory lines (3D7-A10 [reference line; clone of 3D7 derived from NF54], NF54, Dd2-B2 [clone derived from Dd2], and FCB) and 15 African culture-adapted field isolates with naturally occurring *pfcarl* mutations (**Table S2**) were assessed against three antimalarial inhibitors (KAF156, MMV907364 and MMV007564) that are each thought to involve PfCARL as a resistance mediator based on in vitro selections. The IC_{50} and IC_{90} values (mean ± SEM; nM), IC_{90}/IC_{50} ratios (mean ± SEM), and fold-changes (FC) in each of these parameters for each isolate or line compared to the 3D7-A10 reference line are shown.

Parasite isolate/line[a]	N[b]	IC_{50} ± SEM (nM)	IC_{90} ± SEM (nM)	Mean IC_{90}/IC_{50} ± SEM (nM)	IC_{50} FC[c]	IC_{90} FC[c]	IC_{90}/IC_{50} FC[c]
KAF156							
3D7-A10	5	9.0 ± 0.3	23.4 ± 3.2	2.6 ± 0.3	1	1	1
NF54	5	8.9 ± 0.3	22.4 ± 2.3	2.5 ± 0.3	1	1	1
BUS-025	5	9.3 ± 0.3	28.0 ± 2.9	3.0 ± 0.3	1	1.2	1.2
BUS-035	5	8.1 ± 0.2	24.5 ± 1.8	3.0 ± 0.2	0.9	1	1.2
BUS-041	5	8.2 ± 0.4	22.6 ± 2.8	2.5 ± 0.2	0.9	1	1
BUS-043	5	9.3 ± 0.5	23.1 ± 2.8	2.5 ± 0.3	1	1	1
MAS-304	5	10.2 ± 0.6	30.4 ± 1.8	3.0 ± 0.1	1.1	1.3	1.2
MAS-337	5	10.6 ± 0.6	32.3 ± 1.0	3.1 ± 0.1	1.2*	1.4*	1.2
PAT-015	5	10.0 ± 0.6	31.2 ± 1.8	3.1 ± 0.1	1.1	1.3	1.2
PAT-023	5	9.1 ± 0.5	29.8 ± 1.9	3.3 ± 0.1	1	1.3	1.3*
PAT-026	5	7.6 ± 0.1	18.7 ± 2.5	2.4 ± 0.3	0.8**	0.8	1
PAT-033	5	9.3 ± 0.2	27.0 ± 2.0	2.9 ± 0.2	1	1.2	1.1
PAT-038	5	9.1 ± 0.3	26.5 ± 2.3	2.9 ± 0.2	1	1.1	1.1
PAT-045	5	9.4 ± 0.3	30.1 ± 1.2	3.2 ± 0.1	1	1.3	1.3*
PAT-046	5	8.7 ± 0.4	26.8 ± 2.5	3.1 ± 0.2	1	1.1	1.2
TDH-191	5	9.0 ± 0.3	32.6 ± 2.4	3.6 ± 0.2	1	1.4	1.4*
SenP019.04	5	9.6 ± 0.7	28.9 ± 2.0	3.0 ± 0.1	1.1	1.2	1.2
Dd2-B2	5	8.6 ± 0.2	25.5 ± 1.4	3.0 ± 0.1	0.9	1.1	1.2
FCB	5	6.9 ± 0.4	12.8 ± 1.0	1.9 ± 0.1	0.8**	0.5*	0.7
Dd2-CARL[V1103L$]	5	53.1 ± 2.5	206.6 ± 7.0	3.9 ± 0.3	5.9**	8.8**	1.5
MMV907364							
3D7-A10	5	199.0 ± 4.0	470.0 ± 14.8	2.4 ± 0.1	1	1	1

NF54	5 (4)	204.3 ± 12.6	477.1 ± 2.2	2.3 ± 0.2	1	1	1
BUS-025	5 (4)	284.2 ± 14.9	523.5 ± 8.5	1.9 ± 0.1	1.4**	1.1*	0.8*
BUS-035	5 (4)	275.7 ± 5.8	514.8 ± 5.0	2.0 ± 0.1	1.4**	1.1*	0.8
BUS-041	5	224.6 ± 3.7	485.5 ± 1.3	2.2 ± 0.03	1.1**	1	0.9
BUS-043	5 (4)	277.1 ± 9.2	499.5 ± 2.7	1.9 ± 0.05	1.4**	1.1*	0.8**
MAS-304	5 (4)	315.4 ± 13.2	516.7 ± 2.6	1.7 ± 0.1	1.6**	1.1*	0.7**
MAS-337	5 (4)	314.7 ± 5.4	521.2 ± 4.3	1.7 ± 0.1	1.6**	1.1*	0.7*
PAT-015	4	312.1 ± 10.2	590.7 ± 36.8	1.9 ± 0.1	1.6*	1.3*	0.8*
PAT-023	5 (4)	288.2 ± 6.3	535.4 ± 8.0	1.9 ± 0.1	1.4**	1.1*	0.8*
PAT-026	5 (4)	251.4 ± 9.9	496.1 ± 3.7	2.1 ± 0.1	1.3**	1.1*	0.9
PAT-033	5 (4)	256.0 ± 10.8	488.9 ± 5.1	2.0 ± 0.1	1.3*	1	0.8*
PAT-038	5 (4)	223.5 ± 6.1	484.9 ± 3.0	2.3 ± 0.1	1.1*	1	1
PAT-045	5 (4)	242.1 ± 14.1	512.0 ± 1.0	2.2 ± 0.1	1.2	1.1*	0.9
PAT-046	5	231.8 ± 20.3	515.3 ± 11.9	2.3 ± 0.2	1.2	1.1	1
TDH-191	5 (4)	281.1 ± 11.3	508.1 ± 4.7	1.9 ± 0.1	1.4**	1.1*	0.8*
SenP019.04	5 (4)	317.4 ± 10.9	523.7 ± 7.5	1.7 ± 0.1	1.6**	1.1*	0.7*
Dd2-B2	5	167.3 ± 10.9	458.8 ± 13.9	2.8 ± 0.1	0.8	1	1.2
FCB	5	148.1 ± 6.0	417.8 ± 20.0	2.8 ± 0.2	0.7**	0.9	1.2
Dd2-CARL$^{V1103L\$}$	ND						
MMV007564							
3D7-A10	5	1260 ± 51.2	2720 ± 39.5	2.2 ± 0.1	1	1	1
NF54	5	1571 ± 86.3	2986 ± 139.6	1.9 ± 0.1	1.2*	1.1*	0.9*
BUS-025	5	1812 ± 105.0	3950 ± 196.6	2.2 ± 0.1	1.4**	1.5**	1
BUS-035	5	1731 ± 125.2	3413 ± 332.3	2.0 ± 0.1	1.4**	1.3**	0.9
BUS-041	5	1619 ± 56.1	3044 ± 119.8	1.9 ± 0.05	1.3**	1.1*	0.9**
BUS-043	5	1660 ± 74.9	2992 ± 138.9	1.8 ± 0.1	1.3*	1.1*	0.8**
MAS-304	5	2364 ± 56.0	4661 ± 316.1	2.0 ± 0.1	1.9**	1.7**	0.9
MAS-337	5	2162 ± 61.4	4245 ± 146.1	2.0 ± 0.1	1.7**	1.6**	0.9
PAT-015	5	2079 ± 64.3	4249 ± 348.7	2.0 ± 0.1	1.7**	1.6**	0.9
PAT-023	5	1789 ± 56.4	3806 ± 217.6	2.1 ± 0.1	1.4**	1.4**	1
PAT-026	5	1643 ± 80.5	3019 ± 124.4	1.8 ± 0.1	1.3**	1.1*	0.9**

PAT-033	5	1685 ± 94.9	3076 ± 137.1	1.8 ± 0.1	1.3*	1.1	0.8*
PAT-038	5	1546 ± 58.8	3020 ± 119.8	2.0 ± 0.1	1.2**	1.1*	0.9
PAT-045	5	1684 ± 94.2	3440 ± 151.6	2.1 ± 0.1	1.3**	1.3**	1
PAT-046	5	1762 ± 63.0	3344 ± 175.5	1.9 ± 0.1	1.4**	1.2**	0.9
TDH-191	5	1926 ± 101.4	4145 ± 228.9	2.2 ± 0.05	1.5**	1.5**	1
SenP019.04	5	2070 ± 99.9	3751 ± 380.8	1.8 ± 0.1	1.6**	1.4**	0.8*
Dd2-B2	5	1446 ± 56.3	3315 ± 149.4	2.3 ± 0.1	1.1	1.2**	1.1
FCB	5	1100 ± 70.9	2442 ± 157.2	2.2 ± 0.1	0.9	0.9	1.0*
Dd2-CARL[V1103L$]	ND						

[a] All isolates/lines harbor naturally occurring missense mutations in *pfcarl*, *pfat1* and *pfugt*, genes associated with resistance to GNF179 or KAF156, except for the 3D7-A10 control and NF54 parental line, which express the 'wild-type' or 3D7 reference genome haplotypes of these genes.

[b] N, number of independent experiments, each with technical duplicates. For MMV907364, numbers in parentheses indicate the number of independent experiments (each with technical duplicates) used for the mean IC_{90} ± SEM calculations.

[c] FC, Fold-changes in the IC_{50} and IC_{90} values and in the IC_{90} / IC_{50} ratios of all parasite lines compared to the 3D7-A10 control line. Statistical significance (*P*) was determined using two-tailed Mann-Whitney *U* tests (GraphPad Prism 10). $**P \leq 0.01$, $*P \leq 0.05$.

[$] Dd2-CARL[V1103L] is an in vitro evolved clonal line that arose following pressure with GNF179, and which harbors a V1103L missense mutation in the *pfcarl* gene (*52*). This mutant is resistant to both GNF179 and KAF156 and was used as a resistant reference in the KAF156 dose-response assays.

ND, not determined.

Table S5. Amino acid changes in PfMDR1 edited into parasite lines tested in dose-response assays against compounds that select for *pfmdr1* mutations.

Prior to use, all parasite lines were cloned and sequenced only at the locus where the isogenic parent and edited mutant line differ (**Fig. S10**). The amino acid differences at each codon position are highlighted in blue.

	PfMDR1 Haplotype							
**Line**	**86**	**184**	**750**	**784**	**841**	**924**	**1072**	**1075**
NF10	Y	F	A	-	-	-	-	-
NF10$^{A750T\textit{mdr1}}$	Y	F	**T**	-	-	-	-	-
Cam3.II C580Y	N	F	-	S	-	-	-	-
Cam3.II C580Y$^{S784L\textit{mdr1}}$	N	F	-	**L**	-	-	-	-
NF54	N	Y	-	-	M	M	-	-
NF54$^{M841I+M924I\textit{mdr1}}$	N	Y	-	-	**I**	**I**	-	-
Dd2B2	Y/F	Y	-	-	-	-	F	S
Dd2B2$^{F1072L\textit{mdr1}}$	Y/F	Y	-	-	-	-	**L**	-
Dd2B2$^{S1075I\textit{mdr1}}$	Y/F	Y	-	-	-	-	-	**I**

Table S6. Primers used for *pfmdr1* edited lines.

Primer	Sequence	Lab identifier	Description
1	ATATATTCTACTTATTGCTATTGCTATGTTCATTTCAGAAACACTCAAAAACT	p6985	SDM *pfmdr1* I841M Fw
2	AGTTTTTGAGTGTTTCTGAAATGAACATAGCAATAGCAATAAGTAGAATATAT	p6986	SDM *pfmdr1* I841M Rw
3	CTCTTTCTGGTTAGCATGGTTATGTCCTTTTATTTTTGTCCAATT	p6987	SDM *pfmdr1* I924M Fw
4	AATTGGACAAAAATAAAAGGACATAACCATGCTAACCAGAAAGAG	p6988	SDM *pfmdr1* I924M Rw
5	TTTACGTATCATTTATAAAGAAATATTTctATATAAAAAAGATGTTACTATAATTTTCT	p6989	SDM *pfmdr1* S784L Fw
6	AGAAAATTATAGTAACATCTTTTTTATATagAAATATTTCTTTATAAATGATACGTAAA	p6990	SDM *pfmdr1* S784L Rw
7	CAAATAATCGTAATTGTAAAAATACAACAGAAAATGAAAAAGAAGAGAAAGTTCC	p6916	SDM *pfmdr1* A750T Fw
8	GGAACTTTCTCTTCTTTTTCATTTTCTGTTGTATTTTTACAATTACGATTATTTG	p6917	SDM *pfmdr1* A750T Rw
9	GCAGCAAACTTACTAACACG	p7823	Outer flank PCR
10	TCATTTCATAATGCTCTTTCTGG	p7923	Outer flank PCR, sequencing
11	CTTCAAATAATGGAAATGATAATGGATCGG	p9160	Outer flank PCR, sequencing
12	TGCAAAAACTCCGCTTGACATAT	p9161	Outer flank PCR, sequencing

SDM: Site-directed mutagenesis. Mutated nucleotides are shown in red.

Table S7. Dose-response data for lines with edited PfMDR1 mutations.

The in vitro susceptibilities of four laboratory lines (Cam3.II C580Y, NF10, Dd2-B2, and NF54) and five edited lines with single or double PfMDR1 mutations (**Table S5**) were assessed against eight antimalarial inhibitors (ACT-451840, MMV665789, MMV009063, BI-2536, lopinavir, suloctidil, BCH070, MMV665882) that are each thought to involve PfMDR1 as a resistance mediator based on in vitro selections, as well as lumefantrine. The IC_{50} and IC_{90} values (mean ± SEM; nM) for each edited line and their isogenic parent, as well as fold-changes (FC) in each of these parameters, are shown.

Edited parasite line[a]	N[b]	IC_{50} ± SEM (nM)	IC_{90} ± SEM (nM)	Parent N	Parent IC_{50} ± SEM (nM)	Parent IC_{90} ± SEM (nM)	IC_{50} FC[c]	IC_{90} FC[c]
**ACT-451840**								
**Cam3.II C580Y** MDR1 S784L	4	2.1 ± 0.3	5.3 ± 0.4	4	2.6 ± 0.1	6.2 ± 0.6	0.8	0.9
**NF10** MDR1 A750T	4	0.4 ± 0.1	1.0 ± 0.2	4	0.4 ± 0.04	1.0 ± 0.1	1.0	0.9
**Dd2-B2** MDR1 F1072L	5	22.3 ± 1.7	78.3 ± 7.3	4	1.1 ± 0.1	2.8 ± 0.2	19.8*	28.1*
**Dd2-B2** MDR1 S1075I	4	6.2 ± 0.3	23.0 ± 3.6	4	1.1 ± 0.1	2.8 ± 0.2	5.5*	8.2*
**NF54** MDR1 M841I+M924I	4	82.0 ± 12.7	174.0 ± 3.9	4	0.9 ± 0.1	2.2 ± 0.3	90.2*	79.9*
**MMV665789**								
**Cam3.II C580Y** MDR1 S784L	4	35.5 ± 1.0	143.4 ± 17.3	4	77.9 ± 6.6	264.0 ± 23.2	0.5*	0.5*
**NF10** MDR1 A750T	4	10.4 ± 1.0	23.6 ± 0.9	4	12.6 ± 1.9	29.3 ± 3.1	0.8	0.8
**Dd2-B2** MDR1 F1072L	5	7.9 ± 0.6	20.3 ± 0.4	4	28.9 ± 4.1	79.6 ± 2.4	0.3*	0.3*
**Dd2-B2** MDR1 S1075I	4	15.4 ± 1.4	37.4 ± 0.8	4	28.9 ± 4.1	79.6 ± 2.4	0.5*	0.5*
**NF54** MDR1 M841I+M924I	4	56.3 ± 5.6	118.6 ± 15.9	4	56.6 ± 5.4	140.8 ± 14.2	1.0	0.8
**MMV009063**								
**Cam3.II C580Y** MDR1 S784L	4	136.7 ± 11.8	697.8 ± 100.0	4	285.8 ± 24.6	1237 ± 140.1	0.5*	0.6*
**NF10** MDR1 A750T	4	47.1 ± 6.0	139.2 ± 1.8	4	63.7 ± 4.9	138.8 ± 6.4	0.7	1.0
**Dd2-B2** MDR1 F1072L	5	38.2 ± 0.9	138.7 ± 5.2	4	103.2 ± 3.9	295.7 ± 5.7	0.4*	0.5*
**Dd2-B2** MDR1 S1075I	4	55.0 ± 1.8	174.9 ± 8.6	4	103.2 ± 3.9	295.7 ± 5.7	0.5*	0.6*
**NF54** MDR1 M841I+M924I	4	292.8 ± 31.6	567.7 ± 22.4	4	302.9 ± 36.3	572.2 ± 31.9	1.0	1.0
**BI-2536**								
**Cam3.II C580Y** MDR1 S784L	4	57.8 ± 6.5	292.0 ± 15.0	4	167.0 ± 16.3	600.7 ± 134.3	0.3*	0.5*
**NF10** MDR1 A750T	4	36.4 ± 2.0	117.5 ± 15.0	4	50.7 ± 3.6	173.1 ± 20.9	0.7	0.7
**Dd2-B2** MDR1 F1072L	5	308.3 ± 25.6	1160 ± 21.5	4	64.1 ± 10.3	254.0 ± 15.9	4.8*	4.6*
**Dd2-B2** MDR1 S1075I	4	67.8 ± 1.9	253.2 ± 18.8	4	64.1 ± 10.3	254.0 ± 15.9	1.1	1.0
**NF54** MDR1 M841I+M924I	4	114.1 ± 11.5	253.0 ± 28.8	4	120.5 ± 14.8	288.4 ± 27.8	0.9	0.9
**Lopinavir**								
**Cam3.II C580Y** MDR1 S784L	4	1908 ± 130.4	5672 ± 407.1	4	1981 ± 134.8	5928 ± 313.1	1.0	1.0
**NF10** MDR1 A750T	4	1729 ± 169.2	4630 ± 36.4	4	2132 ± 135.4	5794 ± 615.4	0.8	0.8
**Dd2-B2** MDR1 F1072L	4	2323 ± 131.1	7639 ± 292.2	4	1760 ± 95.4	4854 ± 415.4	1.3	1.6*
**Dd2-B2** MDR1 S1075I	4	1955 ± 82.1	4358 ± 292.3	4	1760 ± 95.4	4854 ± 415.4	1.1	0.9
**NF54** MDR1 M841I+M924I	4	3158 ± 247.0	6439 ± 721.0	4	2807 ± 294.3	5366 ± 345.9	1.1	1.2
**Suloctidil**								
**Cam3.II C580Y** MDR1 S784L	4	1122 ± 135.6	3400 ± 604.3	4	1716 ± 323.0	4600 ± 215.1	0.7	0.7

**NF10** $^{\text{MDR1 A750T}}$	4	342.6 ± 51.4	986.3 ± 32.0	4	326.1 ± 20.1	703.3 ± 100.7	1.1	1.4
**Dd2-B2** $^{\text{MDR1 F1072L}}$	5	246.4 ± 14.6	832.5 ± 88.9	4	723.8 ± 58.0	2355 ± 260.4	0.3*	0.4*
**Dd2-B2** $^{\text{MDR1 S1075I}}$	4	232.6 ± 13.9	823.0 ± 67.9	4	723.8 ± 58.0	2355 ± 260.4	0.3*	0.3*
**NF54** $^{\text{MDR1 M841I+M924I}}$	4	2087 ± 20.3	4396 ± 86.0	4	2196 ± 116.4	4813 ± 158.9	1.0	0.9
**BCH070**								
**Cam3.II C580Y** $^{\text{MDR1 S784L}}$	3	32934 ± 2102	58788 ± 1286	3	31425 ± 2487	57741 ± 886.6	1.0	1.0
**NF10** $^{\text{MDR1 A750T}}$	4	2413 ± 263.4	7270 ± 360.7	4	2362 ± 82.1	7568 ± 444.9	1.0	1.0
**Dd2-B2** $^{\text{MDR1 F1072L}}$	4	752.3 ± 42.5	2696 ± 423.2	4	7722 ± 179.9	22702 ± 3232	0.1*	0.1*
**Dd2-B2** $^{\text{MDR1 S1075I}}$	4	15339 ± 1507	39592 ± 4375	4	7722 ± 179.9	22702 ± 3232	2.0*	1.7*
**NF54** $^{\text{MDR1 M841I+M924I}}$	4	27812 ± 3586	45482 ± 1128	4	29744 ± 3595	46534 ± 1058	0.9	1.0
**MMV665882**								
**Cam3.II C580Y** $^{\text{MDR1 S784L}}$	4	13.4 ± 0.6	34.5 ± 2.6	4	33.4 ± 2.9	82.8 ± 8.2	0.4*	0.4*
**NF10** $^{\text{MDR1 A750T}}$	4	17.4 ± 1.3	44.2 ± 4.1	4	12.6 ± 1.4	31.2 ± 2.1	1.4	1.4*
**Dd2-B2** $^{\text{MDR1 F1072L}}$	5	7.1 ± 0.7	18.1 ± 4.1	5	12.6 ± 0.6	31.7 ± 1.8	0.6**	0.6*
**Dd2-B2** $^{\text{MDR1 S1075I}}$	5	8.0 ± 1.0	26.4 ± 3.8	5	12.6 ± 0.6	31.7 ± 1.8	0.6*	0.8
**NF54** $^{\text{MDR1 M841I+M924I}}$	4	16.6 ± 1.7	50.5 ± 3.1	4	14.4 ± 1.5	43.0 ± 5.1	1.2	1.2
**Lumefantrine**								
**Cam3.II C580Y** $^{\text{MDR1 S784L}}$	4	11.1 ± 0.5	28.9 ± 0.6	4	13.2 ± 0.7	31.7 ± 3.1	0.8	0.9
**NF10** $^{\text{MDR1 A750T}}$	4	1.7 ± 0.3	10.8 ± 0.9	4	1.7 ± 0.3	8.9 ± 0.8	1.0	1.2
**Dd2-B2** $^{\text{MDR1 F1072L}}$	4	1.3 ± 0.1	6.2 ± 0.4	4	4.9 ± 1.4	24.7 ± 2.1	0.3*	0.2*
**Dd2-B2** $^{\text{MDR1 S1075I}}$	4	2.7 ± 0.3	16.2 ± 2.6	4	4.9 ± 1.4	24.7 ± 2.1	0.5	0.7
**NF54** $^{\text{MDR1 M841I+M924I}}$	4	5.0 ± 0.6	15.1 ± 1.1	4	13.5 ± 0.7	31.5 ± 3.4	0.4*	0.5*

[a] A T7-based CRISPR/Cas9 strategy (*55*) was used to introduce *pfmdr1* S784L into Cam3.IIC580Y parasites, A750T into NF10 parasites, F1072L/S1075I into Dd2-B2 parasites (*56*), and M841I+M924I into NF54 parasites (*55*).

[b] N, number of independent experiments, each with technical duplicates.

[c] FC, Fold-changes in the IC_{50} and IC_{90} values of all edited parasite lines compared to their isogenic parent. Statistical significance (*P*) was determined using two-tailed Mann-Whitney *U* tests (GraphPad Prism 10). $^{**}P \leq 0.01$, $^{*}P \leq 0.05$.

Table S8. Allele frequency of missense SNVs in *pfatp4* of laboratory lines and field isolates tested in dose-response assays.

Frequencies of *pfatp4* SNVs per isolate tested in dose-response assays[a]

**Gene description**	*pfatp4* (non-SERCA-type Ca2-transporting P-ATPase)										Geographic origin (Year of collection)[f]
**Gene ID**	PF3D7_1211900										
**Exon ID**	1	1	1	1	1	1	1	1	1	1	
**Codon change**	Ggc/Agc	aCt/aAt	aCt/aGt	Caa/Gaa	Caa/Gaa	Ggc/Agc	aaT/aaG	Caa/Aaa	Gga/Aga	Gag/Aag	
**Amino acid change**	G223S	T416N	T507S	Q715E	Q989E	G1031S	N1045K	Q1081K	G1128R	E1146K	
**Pf6 AAF**[b]	0.179	0.000	0.017	0.000	0.007	0.026	0.061	0.449115	0.666	0.003	
**Pf6 sample count**[b]	441	0	48	0	16	70	181	1568	2539	5	
Dd2-2D4[c]	0	0	0	0	0	0	0	0	1	0	Thailand/SE Asia (1980)
KDG012.19	0	0	0	0	1	0	0	1	1	0	Kédougou, Senegal (2019)
KDG051.19	0	0	0	0	0	0	1	0	0	0	Kédougou, Senegal (2019)
KDG080.19	0	0	0	0	0	0	0	1	1	0	Kédougou, Senegal (2019)
P51.02	0	0	0	0	0	0	0	0	1	0	Pikine, Senegal (2002)
Th046.10	0	0	1	0	0	0	0	0	1	0	Thiès, Senegal (2010)
Th127.09	1	0	0	0	0	0	0	0	0	1	Thiès, Senegal (2009)
Th231.08	0	0	0	1	0	1	0	0	0	0	Thiès, Senegal (2008)
Th235.12	1	0	0	0	0	0	0	0	1	0	Thiès, Senegal (2012)
3D7-ATP4:T416N[d]	0	1	0	0	0	0	0	0	0	0	KAE609-pressured mutant (PMID: 27100094)

[a] The in vitro susceptibilities of a set of 10 laboratory lines and culture-adapted field isolates to 12 compounds were measured using 72 h dose-response assays. Whole-genome sequencing was performed in-house using 300 bp paired-end dual index Illumina libraries prepared from freshly extracted parasite genomic DNA at the end of the assay period (see **Methods**). The *P. falciparum* 3D7 reference genome (PlasmoDB, v48.0) was used to identify naturally occurring single nucleotide variants (SNVs) in the tested strains (see **Methods**). Alternate allele frequencies of the discovered SNVs were derived from observed alternate reads / sum of alternate and reference reads.

[b] AAF, global alternate allele frequency in the Pf6 dataset (*39*) as described in **Data S7**, and number of samples from the full Pf6 dataset (n = 7,113) that have total depth ≥ 50 at the site and alternate allele frequency ≥ 0.5.

[c] Dd2-2D4 was cloned from Dd2, which is a multi-drug resistant line originating from W2, an Indochina III/CDC isolate (*80*). Whole-genome sequencing was previously performed on Dd2-2D4, indicating it has a single missense variant in *pfatp4* relative to 3D7, Gly1128Arg (*81*).

[d] 3D7 was derived from NF54, a strain of African origin that was isolated in the Netherlands (*97*). 3D7-ATP4:T416N is a KAE609-pressured resistant clone (*57*).

[f] The Senegalese field isolates have been previously described (*58, 99*). SE Asia, Southeast Asia; year of collection was included if known.

Table S9. Whole-genome sequencing metrics for *pfatp4* SNV profiling of 9 lines tested for susceptibility to compounds that give rise to PfATP4 mutations.

Sample name		KDG012-19	KDG051-19	KDG080-19	P51-02	3D7-ATP4^{T416N}
Total reads		4,862,529	6,980,644	5,593,921	1,756,362	10,356,504
# Mapped reads		4,447,013	6,365,585	5,076,813	1,630,168	9,674,125
Duplication rate		25.91%	30.16%	29.58%	26.45%	41.42%
General error rate		1.77%	1.73%	1.68%	1.59%	0.47%
Mean mapping quality (Phred)		56.27	56.33	56.4	56.73	58.97
Depth of coverage	mean	42.79	61.55	48.47	15.67	97.67
	SD	36.65	52.05	48.32	12.77	75.65
% of PF genome with > x no. reads	1X	97.56%	97.63%	97.63%	96.05%	97.40%
	5X	95.67%	96.25%	95.75%	89.14%	97.06%
	10X	92.60%	94.64%	92.69%	73.43%	96.73%
	20X	82.64%	89.64%	83.39%	30.01%	95.51%
	30X	69.82%	82.19%	72.21%	5.32%	93.08%

Sample name		Th046-10	Th127-09	Th231-08	Th235-12
Total reads		4,577,389	1,996,513	2,035,051	5,264,220
# Mapped reads		4,197,124	1,859,902	1,900,758	4,723,831
Duplication rate		25.80%	26.80%	30.93%	27.62%
General error rate		1.71%	1.53%	1.53%	1.87%
Mean mapping quality (Phred)		56.36	56.81	56.84	56.32
Depth of coverage	mean	40.63	18.16	18.14	44.57
	SD	40.67	12.46	13.45	46.34
% of PF genome with > x no. reads	1X	97.09%	96.20%	96.10%	97.32%
	5X	95.16%	91.72%	91.41%	95.64%
	10X	92.10%	80.71%	80.26%	92.80%
	20X	81.68%	42.52%	42.54%	83.12%
	30X	68.04%	9.93%	9.98%	70.40%

Table S10. Dose-response data for lines with naturally occurring or compound-selected PfATP4 mutations.

The in vitro susceptibilities of Dd2-2D4 [clone derived from Dd2], 3D7-ATP4^{T416N} [KAE609-pressured resistant clone (*57*)], and 8 Senegalese culture-adapted field isolates with naturally occurring *pfatp4* variants (**Table S8**) were assessed against 12 antimalarial inhibitors, six of which have previously yielded *pfatp4* mutations in selections (KAE609, SJ733, MMV665826, MMV020660, MMV011567, GNF Pf-3703) and six of which are clinically used drugs (artemether, piperaquine, amodiaquine, mefloquine, atovaquone, and chloroquine). The IC_{50} (mean ± SEM; nM) and fold-changes (FC) for each isolate or line compared to the Dd2-2D4 reference line are shown.

Parasite isolate/line[a]	N[b]	IC_{50} ± SEM (nM)	IC_{50} FC[c]
**KAE609**			
Dd2-2D4	4	1.2 ± 0.1	1
3D7-ATP4^{T416N}	4	7.1 ± 0.9	6.1*
KDG012.19	3	1.7 ± 0.2	1.4
KDG051.19	4	1.2 ± 0.1	1
KDG080.19	3	1.1 ± 0.1	0.9
P51.02	4	0.9 ± 0.2	0.8
Th046.10	3	1.0 ± 0.1	0.8
Th127.09	3	1.8 ± 0.1	1.5
Th231.08	3	1.3 ± 0.2	1.1
Th235.12	3	1.4 ± 0.2	1.2
**SJ733**			
Dd2-2D4	4	135.2 ± 17.2	1
3D7-ATP4^{T416N}	4	3390 ± 571.4	25.1*
KDG012.19	3	201.7 ± 9.7	1.5
KDG051.19	4	129.8 ± 9.2	1
KDG080.19	3	118.2 ± 17.5	0.9
P51.02	4	85.2 ± 13.7	0.6
Th046.10	3	138.3 ± 3.2	1
Th127.09	3	150.0 ± 7.8	1.1
Th231.08	3	117.6 ± 14.8	0.9
Th235.12	3	142.0 ± 14.4	1
**MMV665826**			

Parasite isolate/line[a]	N[b]	IC_{50} ± SEM (nM)	IC_{50} FC[c]
**Artemether**			
Dd2-2D4	4	9.5 ± 0.3	1
3D7-ATP4^{T416N}	5	8.9 ± 1.1	0.9
KDG012.19	3	7.4 ± 1.3	0.8
KDG051.19	4	9.2 ± 0.7	1
KDG080.19	3	10.0 ± 1.9	1.1
P51.02	4	6.0 ± 1.4	0.6
Th046.10	3	5.9 ± 0.1	0.6
Th127.09	3	7.2 ± 0.9	0.8
Th231.08	3	11.2 ± 1.1	1.2
Th235.12	3	8.5 ± 1.3	0.9
**Piperaquine**			
Dd2-2D4	4	96.9 ± 7.0	1
3D7-ATP4^{T416N}	5	68.5 ± 8.9	0.7
KDG012.19	3	69.2 ± 2.0	0.7
KDG051.19	4	67.7 ± 7.3	0.7
KDG080.19	3	52.2 ± 7.3	0.5
P51.02	4	54.5 ± 6.8	0.6*
Th046.10	3	72.3 ± 10.9	0.7
Th127.09	3	43.0 ± 1.2	0.4
Th231.08	3	79.1 ± 13.4	0.8
Th235.12	3	65.2 ± 9.2	0.7
**Amodiaquine**			

Dd2-2D4	4	1362 ± 200.8	1
3D7-ATP4^{T416N}	4	50475 ± 10418	37.1*
KDG012.19	3	1783 ± 285.9	1.3
KDG051.19	4	1129 ± 94.3	0.8
KDG080.19	3	1040 ± 64.7	0.8
P51.02	4	619.8 ± 50.4	0.5*
Th046.10	3	1058 ± 82.8	0.8
Th127.09	2	2150 ± 60.0	1.6
Th231.08	2	1465 ± 5.0	1.1
Th235.12	2	1515 ± 75.0	1.1
**MMV020660**			
Dd2-2D4	3	1927 ± 173.2	1
3D7-ATP4^{T416N}	4	44100 ± 6251	22.9
KDG012.19	3	3233 ± 519.8	1.7
KDG051.19	4	2288 ± 373.3	1.2
KDG080.19	3	1930 ± 172.1	1
P51.02	4	1171 ± 177.2	0.6
Th046.10	3	2037 ± 163.7	1.1
Th127.09	3	2660 ± 285.8	1.4
Th231.08	3	1797 ± 288.3	0.9
Th235.12	3	2093 ± 357.1	1.1
**MMV011567**			
Dd2-2D4	4	1179 ± 236.7	1
3D7-ATP4^{T416N}	4	13695 ± 1452	11.6*
KDG012.19	3	1703 ± 253.9	1.4
KDG051.19	4	1029 ± 94.4	0.9
KDG080.19	3	809.0 ± 83.4	0.7
P51.02	4	619.8 ± 107.1	0.5
Th046.10	3	938.7 ± 62.4	0.8
Th127.09	3	2283 ± 128.4	1.9
Th231.08	3	1049 ± 166.2	0.9
Th235.12	3	1625 ± 424.6	1.4
**GNF Pf-3703**			

Dd2-2D4	4	13.0 ± 0.2	1
3D7-ATP4^{T416N}	5	8.1 ± 1.3	0.6*
KDG012.19	3	6.3 ± 1.5	0.5*
KDG051.19	4	7.5 ± 1.0	0.6*
KDG080.19	3	5.3 ± 0.4	0.4*
P51.02	4	8.5 ± 1.3	0.7*
Th046.10	3	5.6 ± 0.6	0.4*
Th127.09	2	4.3 ± 0.3	0.3
Th231.08	3	10.5 ± 1.9	0.8
Th235.12	3	6.0 ± 1.2	0.5*
**Mefloquine**			
Dd2-2D4	4	24.1 ± 3.3	1
3D7-ATP4^{T416N}	5	19.6 ± 1.0	0.8
KDG012.19	3	28.8 ± 2.0	1.2
KDG051.19	4	33.1 ± 3.4	1.4
KDG080.19	3	34.4 ± 2.4	1.4
P51.02	4	5.3 ± 1.3	0.2*
Th046.10	3	25.4 ± 5.0	1.1
Th127.09	3	31.1 ± 1.8	1.3
Th231.08	3	20.3 ± 2.7	0.8
Th235.12	3	23.4 ± 0.3	1
**Atovaquone**			
Dd2-2D4	4	0.4 ± 0.1	1
3D7-ATP4^{T416N}	5	0.7 ± 0.2	1.6
KDG012.19	3	0.3 ± 0.0	0.6
KDG051.19	4	0.3 ± 0.0	0.8
KDG080.19	3	0.1 ± 0.0	0.3
P51.02	4	0.3 ± 0.0	0.6
Th046.10	3	0.3 ± 0.1	0.7
Th127.09	3	0.2 ± 0.0	0.5
Th231.08	3	0.2 ± 0.0	0.5
Th235.12	3	0.2 ± 0.0	0.5
**Chloroquine**			

Dd2-2D4	4	2070 ± 149.8	1
3D7-ATP4^{T416N}	4	49200 ± 3224	23.8*
KDG012.19	3	3517 ± 143.1	1.7
KDG051.19	4	2158 ± 56.2	1
KDG080.19	3	1603 ± 294.2	0.8
P51.02	4	1026 ± 163.5	0.5*
Th046.10	3	2150 ± 49.3	1
Th127.09	2	2520 ± 220.0	1.2
Th231.08	2	1380 ± 250.0	0.7
Th235.12	3	2440 ± 435.5	1.2

Dd2-2D4	4	292.5 ± 41.5	1
3D7-ATP4^{T416N}	5	16.4 ± 2.0	0.1*
KDG012.19	3	21.1 ± 3.1	0.1
KDG051.19	4	22.1 ± 2.4	0.1*
KDG080.19	3	22.5 ± 1.4	0.1
P51.02	4	218.5 ± 29.3	0.7
Th046.10	3	21.3 ± 2.4	0.1
Th127.09	3	17.4 ± 2.5	0.1
Th231.08	3	185.7 ± 9.9	0.6
Th235.12	3	22.7 ± 3.6	0.1

[a] All isolates/lines harbor naturally occurring missense variants in *pfatp4*.

[b] N, number of independent experiments, each with technical triplicates.

[c] FC, Fold-changes in the IC_{50} values of all parasite lines compared to the Dd2-2D4 reference line. Statistical significance (P) was determined using two-tailed Mann-Whitney U tests (GraphPad Prism 10). $*P \leq 0.05$.

Data S1. In vitro compound-selected clone metadata and sequencing statistics.

Attached Excel spreadsheet. Characteristics of compound-selected clones analyzed in this study and whole genome sequencing statistics.

Data S2. Compound library description and IC_{50}s across parasite life stages.

Attached Excel spreadsheet. Characteristics of compounds used to select for the resistant clones analyzed in this study.

Data S3. High-quality single nucleotide variants (SNVs) and indels in compound-selected clones.

Attached Excel spreadsheet. SNVs and indel mutations identified in this study.

Data S4. Copy number variants (CNVs) in compound-selected clones.

Attached Excel spreadsheet. CNVs identified in this study.

Data S5. Compounds classified based on target or resistance gene evidence.

Attached Excel spreadsheet. List of compounds with indication of target or resistance gene evidence, if applicable.

Data S6. Characteristics of SNVs in 4,491 core genes with at least one SNV in compound-evolved clones or in Pf6 field samples.

Attached Excel spreadsheet. Statistics on numbers of synonymous and nonsynonymous SNVs per gene among compound-selected clones or the Pf6 dataset of field isolates.

Data S7. Prevalent field SNVs in *pfcarl*, *pfmdr1*, *pfcytb* and *pfatp4*.

Attached Excel spreadsheet. Prevalent SNVs in Pf6 dataset of field isolates for selected genes.
